# Segmental duplications in the silkworm genome

**DOI:** 10.1186/1471-2164-14-521

**Published:** 2013-07-31

**Authors:** Qian Zhao, Zhenglin Zhu, Masahiro Kasahara, Shinichi Morishita, Ze Zhang

**Affiliations:** 1 Laboratory of Evolutionary and Functional Genomics, School of Life Sciences, Chongqing University, Chongqing 400044, China; 2 Department of Computational Biology, Graduate School of Frontier Sciences, The University of Tokyo, Kashiwa 277-0882, Japan

## Abstract

**Background:**

Segmental duplications (SDs) or low-copy repeats play important roles in both gene and genome evolution. SDs have been extensively investigated in many organisms, however, there is no information about SDs in the silkworm, *Bombyx mori*.

**Result:**

In this study, we identified and annotated the SDs in the silkworm genome. Our results suggested that SDs constitute ~1.4% of the silkworm genome sequence (≥1 kb in length and ≥90% in the identity of sequence); the number is similar to that in *Drosophila melanogaster* but smaller than mammalian organisms. Almost half (42%) of the SD sequences are not assigned to chromosomes, indicating that the SDs are challenges for the assembling of genome sequences. We also provided experimental validation of large duplications using qPCR. The analysis of SD content indicated that the genes related to immunity, detoxification, reproduction, and environmental signal recognition are significantly enriched in the silkworm SDs.

**Conclusion:**

Our results suggested that segmental duplications have been problematic for sequencing and assembling of the silkworm genome. SDs may have important biological significances in immunity, detoxification, reproduction, and environmental signal recognition in the silkworm. This study provides insight into the evolution of the silkworm genome and an invaluable resource for insect genomics research.

## Background

Genome sequencing provides the opportunity to assess fundamental biological processes of genome evolution [1]. With the increasing of finished genome sequences, the field of genome evolution is experiencing a renaissance of activity and many questions of genome architecture as well as genome evolution are resolved using computational studies. However, the identification and characterization of highly homologous sequences in the genome remain problematic. Segmental duplications (SDs), defined as low-copy repeats of DNA segments (blocks of sequence ≥ 1 kb in length and showing ≥ 90% sequence identity), are a class of homologous sequences. Since SDs are hotspots of copy number variance (CNV) as well as pools of gene innovation and disease-causing rearrangement [2-15], they have long been regarded to be involved in functional redundancy, adaptive evolution, and structure dynamics of chromosomal evolution. Thus, identification and annotation of SDs are important for understanding the structure and evolution of a genome.

Up to now, although the analyses of SDs have been done in many organisms whose genome sequences were completed [2-11], no analysis has ever been performed in the domesticated silkworm, *Bombyx mori*. The silkworm genome sequence has been released [16,17] and the amounts of hierarchical bacterial artificial chromosome (BAC) data are available, this provides us an opportunity to identify and annotate SDs in the silkworm genome. In this study, we used two computational methods to identify the SDs. The first one, named whole-genome assembly comparison (WGAC), is a BLAST-based approach that performs an all-by-all comparison of assembled genome sequence [18]. The second one, whole-genome shotgun detection (WSSD), develops a model for distinguishing unique and duplicated sequence on the basis of the depth of coverage after whole-genome shotgun sequence reads were aligned to a reference genomic segment [4]. Duplication regions would display a higher reads depth than depths-of-average. Experiments (real time fluorescent quantitative PCR (q-PCR)) have been used to validate these large duplication sequences [19-21]. Here, we present a set of the silkworm SDs that provides a framework for future evolutionary study. In addition, this resource also provides invaluable information in finishing the silkworm genome.

## Results

### Genome-wide identification of the silkworm SDs

**Figure 1:**
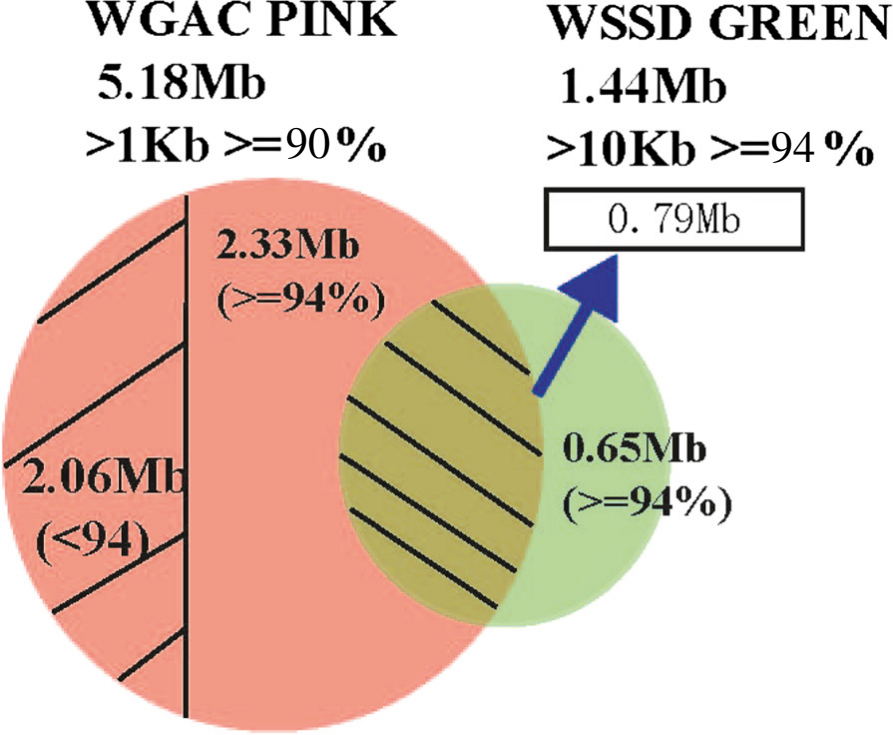
**The comparison of silkworm SDs identified by WGAC and WSSD pipelines.** There are 5.18 Mb (orange) and 1.44 Mb (Green) putative duplications predicted by WGAC and WSSD algorithms, respectively. The overlapped region was SDs predicted using both analyses. The shaded orange was SDs defined by WGAC with sequence identity less than 94%.

We used two well-established computational methods, whole genome assembly comparison (WGAC) and whole genome shotgun sequence detection (WSSD), to detect putative SDs in the silkworm genome. The central aspect of WGAC is to generate a compact version of the silkworm genome sequence by firstly removing high-copy repeats from the genome using RepeatMasker (http://www.repeatmasker.org/). This pipeline has two advantages: (1) the BLAST search is faster because of the overall reduction in sequence content; (2) it enhances the ability to detect duplications riddled with high-copy repeats that would be missed. Remarkably, we identified 5.17 M or 73937 pairwise alignments as duplications by WGAC analysis (≥90% identity and ≥1 kb in size) (Figure 1). And about 42% of these duplications were mapped to the unassigned chromosome – ChrUn.

We also applied the WSSD strategy separately to the silkworm genome (432 Mb) and BACs (8.2 Mb) sequences. This step is to map 3.8 million reads against both the genome sequence and BACs data to assess the reads depth (RD) and divergent read ratio in 5-kb windows sliding in 1 kb steps (see Methods). We found that there were 12 large duplication segments in BACs and 117 in the genome (≥94% identity and ≥10 kb in size; Figure 1; Table 1). Like the SDs in *Drosophila* genome [22], the silkworm genome seems to be significantly poor in large block (>10 kb; Figure 1).

**Table 1:** q-PCR validation of a subset of WSSD duplications in BACs

			**WSSD duplicated regions**		
**Accession**	**BAC clone**	**Chr. #**	**Length**	**Read depth (#/5 kb)**	**Q-PCR result**
AB159446.1	559G11	chromosome 11	12750	3535.2	+
AP009014.1	047D02	chromosome 11	15001	753.3	-
AP009015.1	048C11	chromosome 17	14108	6076	+
AP009017.1	503G12	chromosome 3	11780	2365.9	+
AP009018.1	503 L14	chromosome 6	36001	1235.2	+
AP009021.1	513O16	chromosome 22	14923	2649.9	+
AP009022.1	513P13	chromosome 23	13726	711.8	+
AP008992.1	001D20	chromosome 27	11648	906	+
AP008996.1	006H21	chromosome 8	19541	629.1	+
AP009013.1	041P16	chromosome 4	131526	633.4	+
AP009006.1	019 F14	chromosome 1	11763	1249.9	+

In this study, we totally detected 6.6 Mb SDs in *B. mori*, which cover ~1.4% of the silkworm genome (6.6 Mb/432 Mb; Figure 1), size ranging from 1 kb to 23 kb (Additional file 1). Previous studies suggested that high-identity duplications (identity > 94%) frequently collapsed within working draft sequence assemblies [23] and may represent artificial duplications within an assembly [18]. We compared the WGAC results to those detected by WSSD approach and found that 45.1% of the SDs identified by WSSD were not detected by WGAC, which may be caused by collapsed duplications (Figure 1). In addition, we also found that 0.79 Mb of the duplications detected by WGAC were also detected by WSSD, and these are the high-confidence SDs in the silkworm genome.

**Figure 2:**
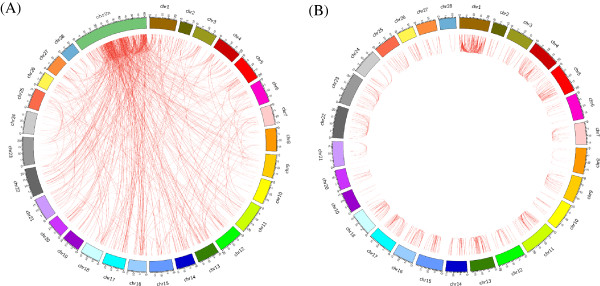
**Silkworm SDs and its paralogous regions in the genome. (A)** SDs in the silkworm (only one paralogous region of SDs was shown). **(B)** Patterns of intrachromosomal segmental duplications.

**Figure 3:**
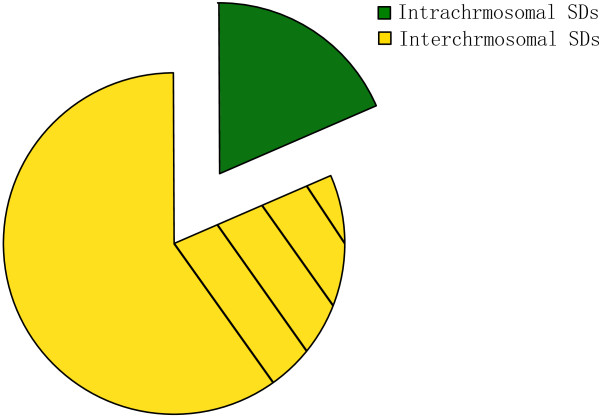
**Sequence property of the silkworm SDs.** The proportions intrachromosomal and interchromosomal duplications are shown. The shaded yellow region represented the SDs whose paralogous sequences distributed on ChrUn.

Our results showed a bias toward interchromosomal duplications compared to intrachromosomal alignments (Figure 2), which greatly differs from previous observations [6,22,24]. However, this is not enough for us to make a conclusion that the silkworm genome had a bias for interchromosomal duplication since about 39.5% of these interchromosomal duplications were assigned to the ChrUn (Figure 3; Additional file 2). We noted that a fraction of the silkworm genome sequence (~60 Mb, 13% of the genome) has not been assigned to the chromosomes, which is about six silkworm chromosomes long and relatively larger compared to other sequenced genomes [4,6,24]. In this study, we treated the unmapped sequences as a separate chromosome, ChrUn. The intrachromosomal duplications can be further categorized as tandem duplications and interspersed duplications. We found that there were about 29.5% of intrachromosomal duplications (*n* = 450) mapped within 1 Mb of one another and within these duplications, the majority of them were tandem duplications with no one gene separating them.

### Patterns of the silkworm SDs

**Figure 4:**
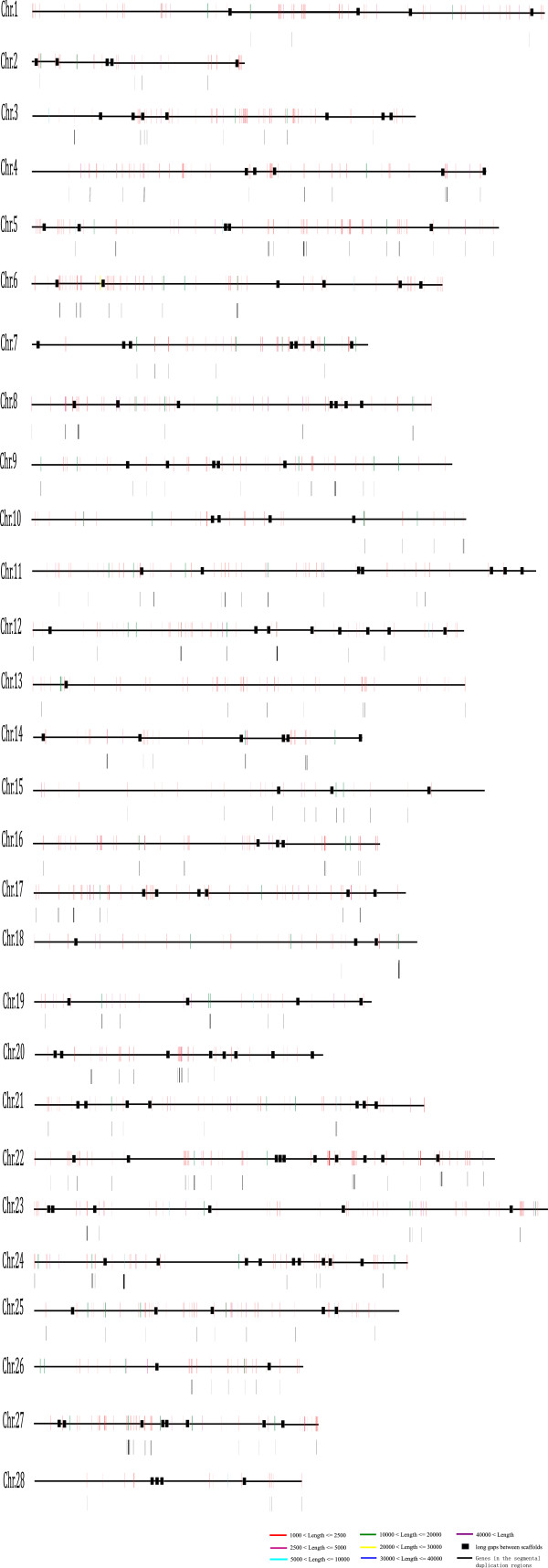
**Silkworm SDs for each chromosome.** Different color bars represented different lengths of SDs. The large gaps between each scaffold were shown in black rectangle. Genes embedded in each SDs were shown as black bars located under each chromosome.

The distribution of SDs on chromosomes (not including ChrUn) is largely nonrandom (Figure 4). Firstly, as expected, the “uncharacterized chromosome” (ChrUn), which can not be uniquely mapped to the genome, contained the majority of SDs (~42%; Figure 3; Additional file 2). And different chromosomes contained various SDs contents (Figure 2; Figure 4). Chromosomes 1, 5, 22 and 27 had the highest SDs densities (Figure 2; Figure 4) with >1.5 folds of the duplication content of the genome average (unplaced contigs were excluded), while the values for chromosomes 8, 19, 20 were much less than half of those (Figure 2; Figure 4). Besides, previous studies demonstrated that SDs are enriched in pericentromeric and subtelomeric regions [22,24]. Although the silkworm chromosomes are holocentromere, we did find increased contents of SDs in some regions along some chromosomes (*p*-value < 0.05, Chi-square tests) (Figure 4; Additional file 3). Further survey showed that some gene families are enriched in these SDs, such as odorant receptor gene cluster, ras-related protein and alkaline phosphatase gene cluster, which is basically consistent with the findings in *Drosophila melanogaster*[22].

Among duplication segments, there is a class of large tracts, termed as “duplication blocks” (if some other SDs were identified within 100 kb from the coordinates of a SD, this whole large region is termed as a duplication block and gaps were excluded) [25]. We found that such duplication blocks contained protein-coding genes (Figure 4). The SDs are distributed near the gaps (within 1 Mb of the gaps) of the reference genome sequence for some chromosomes, for example, chromosomes 6, 14, 17 and 27 (45.5%-83.3%), indicating that these gaps themselves would be high-copy duplications. Furthermore, our results showed that a large proportion of SDs in the silkworm genome were on ChrUn. Thus, probably SDs may be the problems of the silkworm genome assembling.

### Sequence properties of the silkworm segmental duplications

**Figure 5:**
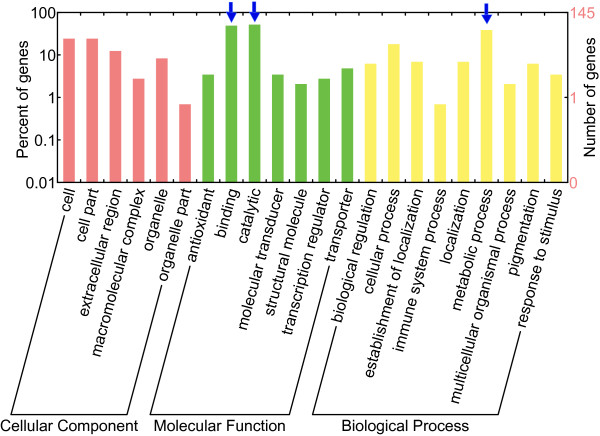
**GO annotation of the genes in SDs.** Gene classes overrepresented in SDs were indicated by blue arrow. In molecular function, binding and catalytic were much higher than other categories (**p < 0.01, T-test). In biological process, metabolic process was much higher than other categories (*p < 0.05, T-test).

We analyzed the composition of genes in the SDs. In total, 320 putative genes were identified in the SD regions. Among these 320 genes, 304 were located in the SDs identified by WGAC, while only 65 were in SDs identified by WSSD. 49 genes were overlapping between the two methods. Besides, 50% (159/320) of the silkworm segmental duplication intervals identified by WGAC and WSSD contain gene duplicates (Additional file 4). Although functions of some genes are unknown or hypothetical, a large proportion of 320 genes belong to multigene families, such as *Lipoprotein receptors, Histone* and *P450s* (Additional file 5). In order to test the hypothesis that particular gene classes were overrepresented in the SDs [24], we used Gene Ontology (GO) to annotate all the genes. The genes with the functions of binding, catalytic and genes related to metabolic process were enriched in the SDs (Figure 5). Pfam was also used to predict the functions of genes in the SDs and the results showed a similar trend (Additional file 6).

On the basis of annotated functions, the genes in the SDs are classified into three categories. The first category includes the genes associated with detoxification (i.e. cytochrome P450); the second one contains the genes related to innate immune response (i.e. serine protease) and the last category includes the genes with functions of environmental signal recognition (i.e. olfactory receptor) (Table 2). Indeed, a previous study suggested that as many as 50 cytochrome P450 genes were present in gene cluster on chromosomes and 78 cytochrome P450 genes were functional in the silkworm genome [26]. Recent studies revealed that the glucose-methanol-choline (GMC) oxidoreductases and 30 K proteins (30 KPs) families experienced lineage-specific expansions in the silkworm [27,28]. For the GMC gene family, two members (BGIBMGA012997-TA and BGIBMGA012998-TA) of the GMC *β* subfamily which made a major contribution to expansion of the silkworm GMC genes are located in the SDs. Furthermore, such lineage-specific GMC *β* subfamily expansion was associated with immunity [27]. In addition, there is a lepidopteran-specific Lipoprotein_11 family in the silkworm, whose members were involved in various physiological processes such as energy storage, embryomic development and immune response [28]. We identified 9 lepidopteran-specific Lipoprotein_11 genes in the SDs. These results showed that SDs might play important roles in the evolution of the silkworm lineage-specific functions.

**Table 2:** Repertoires and evolutionary mechanisms of selected duplicated genes or gene families in mammals and silkworm

	**Human**	**Fly**	**Silkworm**	**Mechanisms**
Cytochrome P450 enzyme	57	86	84	catalyze the oxidation of organic substances
Ras subfamily	27	> = 3	> = 3	participate as central control elements in signal transduction pathways
Serine protease	86	147	51	variety of physiological processes, such as cell signaling, defense and development
Glucose-methanol-choline oxidoreductase (GMC)	1	15	43	Developmental or physiological process, immunity
Olfactory Receptor	1152	14	66	responsible for the detection of odor molecules
30 K proteins (30KPs, Lipoprotein_11)	-	-	73	physiological processes such as energy storage, embryonic development, and immune response

We also analyzed transposable elements (TEs) composition by comparing SDs to the sequences drawn nearby with identical sizes (Table 3, Methods). Strikingly, we found that the content of short interspersed elements (SINEs) in the SDs is much lower than the genome average (2.57% vs. 12.8%). And, SINE content increased when SDs’ flanking sequences were taken into consideration (Table 3). An opposite trend was observed with respect to DNA transposons and long terminal repeat (LTR) retrotransposons. Unlike segmental duplications in human, which were rich in SINE [12], the silkworm SDs were characterized by enrichment of DNA transposons and LTR retrotransposons (Table 3). A similar trend was also observed in the flanking regions of SDs. The high TE enrichment in SDs suggests a potential implication of repeats in SD formation, as described previously [22].

**Table 3:** Repeat properties of the silkworm genome, duplication, and flanking region

**Repeat**	**Duplication**	**%**	**2.5-kb flanking region**	**%**	**Genome**	**%**	**Enrichment in duplication content**
**DNA**	**264496**	**3.85**	**532045**	**3.29**	**13080647**	**3.0**	**1.28**
Non-LTRs	346036	5.04	1258918	7.77	59494107	13.8	0.644
SINE	176716	2.57	523426	3.24	55,380,558	12.8	0.20
**LTR**	**166280**	**2.40**	**657375**	**4.06**	**7,130,669**	**1.7**	**1.41**
Other	51010	0.74	113269	0.699	31050702	7.2	0.10
Total bp analyzed	6 Mb		16.19 Mb	49.03	153056036	35.4	

The repeat contents of three regions of the silkworm genome were compared: duplicated regions as detected by whole-genome analysis comparison; 2.5-kb flanking regions immediately flanking the clustered duplications and the genome average. Enrichment was defined as the repeat content of duplicated sequences divided by the repeat content of unique sequences.

### Experimental validation of a subset of SDs

**Figure 6:**
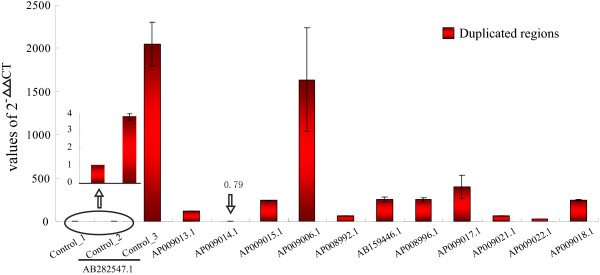
**qPCR confirmation.** X-axis shows the different BAC clones while Y-axis represents the relative quantification (RQ) value of the reference region.

SDs, defined as low-copy repeats of genome segments, can be detected by qRT-PCR-based copy number screening [21]. By qRT-PCR, we validated the SDs in 11 BACs that were determined by WSSD strategy (Table 1). The lengths of these SDs in the BACs range from 11 kb to 13 kb and the corresponding reads depths are listed in Table 1. Previous studies showed that the copy number of SDs should be less than that of TEs [22]. Our qRT-PCR and reads mapping results both confirmed this (Figure 6, Additional file 7), except for the BAC-AP009006.1 (Figure 6). This BAC clone contained a SD whose qPCR result is similar to the TEs. We examined the sequences of this SD and found that this SD contained a large *CR1* transposable element (NonLTR). We aligned them with the repeat database and the identity was less than 90%. Consequently, we did not mask this region by RepeatMasker (the cutoff we set before, see Methods).

The SDs in (91%, 10/11) BACs were confirmed to be positive duplications by qRT-PCR (Figure 6). It should be emphasized that not all true duplications could be detected by qRT-PCR, especially low-copy duplications with divergent reads ratio > 0.8 are difficult to be detected. Thus, 9% for false positive rate is a conserved estimate in our WSSD strategy.

## Discussion

### Quality of SD detection

SDs have been extensively studied in many organisms including vertebrates and invertebrate [5,6,8,18,22,24,25,29]. Here we performed a systematic analysis of segmental duplication in the silkworm genome using two different approaches, a sequence assembly-based approach (WGAC) and a whole-genome shotgun sequence detection method (WSSD). The power of SD detection depends largely on the quality of the underlying sequence assembly and strategy used. There are four factors that would influence the detection of SDs in genome assembly: (1) depth of genome sequencing, (2) methodology of assembly, (3) quality of genome annotation and (4) level of allelic variation. In order to take advantage of low level of allelic variation, we implemented a modification of WSSD approach described before which is entailed a quality assessment of underlying reads to calculate percent identity and determine the proportion of variants within a certain region in a genome [6].

It should be noted that there are some limitations in this study. On one hands, the number of SDs may be underestimated. For example, regions of extremely high sequence identity may collapse during assembly, which may result in the underestimation of fraction of genome showing relatively high identity. That is why some highly homologous gene families, such as carboxylesterases [30], were not detected in our study, although other highly homologous gene families were detected (i.e., cytochrome P450 genes, serine protease, histones). Besides, the power of SD analysis depends largely on genomic sequence and its annotation. The presence of sequence gaps as well as contig orientation may influence the detection of SDs in a genome. The current silkworm genome sequence only covers about 85% of the genome size and has many gaps. Thus, this study may underestimate the SD content in the silkworm. On the other hand, there may be some false positives in the SDs identification using WSSD. This may be due to the incomplete annotation of repeats in the current silkworm genome. In order to get the accurate information about the large SDs and exclude false positive cases, further annotation of repeats as well as FISH hybridization are needed in further study. In the silkworm genome, about 85% of the SDs are shorter than 2 kb. This suggests that SDs in the silkworm are much smaller that in mammals, which is consistent with other invertebrates such as *D. melanogaster*[22]. Thus, PCR validation would be more favorable.

Despite these limitations, some other important trends regarding the SDs in the silkworm appeared. Our estimation of the SD content is consistent with that in *D. melanogaster*[22] but much lower than mammals [6,15,24] (Additional file 8). We proposed that this difference may be due to biological reasons to be investigated. A previous study also supposed that SDs in invertebrates are much less than that in vertebrates [31].

Based on a new assembly of the silkworm genome [32], we found that the SDs were distributed in a nonuniform fashion across the genome (Figure 4). For example, there are some SD enrichments on chromosomes (Additional file 3). And there are some SDs that reside within 1 Mb of the “gaps” on chromosomes (Chrs 6, 14, 17 and 27) (Figure 4), suggesting that SDs may be the problematic regions for both clone-based and whole-genome shotgun sequencing methods.

### Enlightenment for genome assembling

The published silkworm genome sequence represents one of the first attempts to sequence and assemble a lepidopteran genome mainly based on shotgun sequencing read data. One of the greatest challenges of genome assembly lies in the segmental duplications, because of high degree of sequence identity comparing with each other [31,33-35]. There are three possibilities when SDs are encountered during sequencing and assembling: (1) these SDs may be recognized distinctly and resolved properly; (2) because of the presence of virtually identical sequence reads in the database, the sequences may be underrepresented and (3) SDs may be mistakenly assembled into the genome. The second and the third outcomes create numerous gaps [31]. Thus, genome-wide studies of segmental duplication contents become an effective measure to assess the quality of whole-genome sequence assemblies [36] and provide important information for the users of genome sequence.

There are a few important conclusions drawn from this study with respect to genome assembling. The complex, highly duplicated nature of SDs is not amenable to high-throughput assembly methods without refinement. For example, some whole-shotgun sequence approach, such as *Arachne*, would collapse highly identical duplications [37]. Currently, three types of gaps are recognized within the working draft sequence [31,38]. The first type, named as trivial gaps, is no more than 100 bp in length. Gaps between ordered clones or sequence contigs are the second type, which is easily closed by sequencing of bridging clones obtained from pair-end sequence data. However, the third type is more complicated because it is associated with SDs. The solution for this kind of gaps is difficult because we should recognize the SDs first in the genome. Some gaps in the silkworm genome belong to this type, since some SDs are distributed in the flanks of these gaps (Figure 4). The “unplaced” chromosome (ChrUn) showed a significant enrichment for SDs (Additional file 2), with almost 42% of the duplications assigned to ChrUn. Further efforts should target on these regions if we want to get the better sequence of the silkworm genome. Figure 1 showed the comparison of SDs detected by WSSD and WGAC and the results suggested that 9.82% of SDs could only be detected by WSSD. If we use the experimental qRT-PCR data to estimate false positive rate (9%), we conclude that 0.065 Mb SDs have not been resolved within the genome. Thus, our results suggest that, at present, clone-ordered-based approaches for sequence assembly appear to be a more effective resolution for identifying the true locations, organization and complexity of SDs. Furthermore, the intrachromosomal SDs are comparatively less based on the current silkworm genome assembly. Two reasons would contribute to this: (1) as many as 39.5% of interchrmosomal duplications were found to have paraloguous sequences on ChrUn. The gaps on the chromosome might lead to underestimate of the intrachromosomal SDs; (2) the silkworm genome has some distinctive features: there are 28 chromosomes while the genome is only about 432 Mb. The chromosome sizes are relatively small (about average 15.4 Mb for each chromosome); and TEs content is large in the genome (~35%). There is another possibility. Because of short chromosomes, intrachromosomal duplications are so few. A previous study showed that interchromosomal duplications are shorter (median length 2.5 kb) while intrachromosomal duplications are much larger (median length 20 kb) in the bovine genome [24]. However, the silkworm genome is lack of large duplications and most of the duplications were less than 2.5 kb.

### SD content analysis

The correct assembly of SDs is not considered to be high priority, especially the draft phase of a genome sequence, due to the gene-poor content of such regions [6]. However, in some organisms, such as human, highly segmental duplications (~6%-7%) were rich in TEs and genes [12]. A similar pattern is also found in the silkworm. The gene content in the silkworm SDs occupies about ~2% of the genome but the SDs constitute only 1.4% of the genome sequence. In addition, some TEs were enriched in the SDs, such as DNA transposons and LTR retrotransposons (Table 3). Comparing with other insects (e.g. fruit fly), the silkworm genome harbors a lot of TEs, about 35% of the genome [39] and LTR retrotransposons are the most common TEs in *B. mori*[40]. Thus, TEs could be involved in the formation of SDs in the silkworm. Besides, many duplicated genes and gene families were found to reside in the SDs and some of them were implied in lineage-specific adaptations of organisms to a particular environment. Antimicrobial peptide (AMPs) genes, which play important roles in innate immune system in insects [41], were found to be enriched in the silkworm SDs (Additional file 6). Some of GMC genes, which expanded in the silkworm and associated with immunity, were also found in the SDs. The members of the lepidopteran-specific Lipoprotein_11 family and serine protease gene family related to immune response were enriched in the SDs [42]. Furthermore, since frequently encountered a wide variety of secondary products in the mulberry leaves, such as plant allelochemicals, the silkworm has evolved special enzymes to adapt to the digestion of secondary products in mulberry leaves [26,43]. For example, cytochrome P450 enzymes are involved in such biological processes in the silkworm. In this study, we found that some members of cytochrome P450 gene family are located in the silkworm SDs. Besides, some genes which were involved in silk production were also found in SDs, such as proteasome. In this sense, SDs may play important roles in the evolution of species specific functions.

There are some practical and biological implications for the identification of genes in SDs. Previous studies showed that SDs are candidates for the evolution of organism-specific genes [44,45]. Some gene families under selection in vertebrates were identified, such as cytochrome P-450, olfactory receptor [46,47]. However, the functions of many genes in the silkworm SDs are still unclear on the basis of BLASTP searching against *nr* databases. We used these unannotated genes located in SDs as references to search against the protein sets of related insects, especially Lepidopteran species. We found that most of these unannotated genes had orthologs in other insects, especially in Lepidoptera (Additional file 9). For example, BGIBMGA003910-PA, which is poorly annotated in the silkworm database, has orthologus in other insects (such as monarch butterfly, *Danaus plexippus, Heliconius melpomene*, *Dendroctonus ponderosae, Nasonia vitripennis*), but the identity was much higher in Lepidoptera (Additional file 10). The silkworm is an important economic insect and it is also the model organism for molecular genetic and genomic studies of order Lepidoptera [48]. Our study presented invaluable information for the SDs in the silkworm, which facilitates understanding the evolution of the silkworm genome as well as the biology of the silkworm.

## Conclusion

We for the first time analyzed the SDs in the silkworm genome and found that SDs constitute ~1.4% of the silkworm genome sequence (≥1 kb in length and ≥90% in the identity of sequence). This number is similar to that in *D. melanogaster* but smaller than mammalian organisms. Almost half (42%) of the SD sequences are not assigned to chromosomes, suggesting that the SDs are challenges for the assembling of genome sequences. Large duplications were also validated by qPCR experiments. The genes related to immunity, detoxification, reproduction, and environmental signal recognition are significantly enriched in the silkworm SDs, implying that SDs may have important biological significances in the above physiological processes. Our results provide insight into the evolution of the silkworm genome and an invaluable resource for insect genomics research.

## Methods

### Genome resources

We downloaded the silkworm genomic sequence (9×) from the silkworm genome database (SilkDB, http://silkworm.genomics.org.cn/) and the whole genome shotgun sequence (WGS) reads from [49]. The source of the BAC library DNA was NCBI http://www.ncbi.nlm.nih.gov/. This BAC library contained 46 clones which are distributed in 22 chromosomes, representing 1.8% of the silkworm genome.

### Whole-genome alignment comparison (WGAC)

We performed a combination of sequence analysis software and a list of Perl scripts to optimize the detection of large segmental duplications (length ≥ 1 kb and identity ≥ 90%) [6].

The large contigs in the silkworm genome were broken into tractable 400 kb segments. Using RepeatMasker (Smit and Green http://www.repeatmasker.org/, version open-3.3.0), we identified the high-copy repeats. The silkworm genome is rich in TEs (~35%) [39]. We used our own TE dataset as repeat database (http://gene.cqu.edu.cn/BmTEdb/) in the running of RepeatMasker. These reference contigs were masked at 10% divergence level from TEs. After that, all these high-copy repeats were deleted out of the sequences. The resulting unique genome DNAs then underwent global BLASTN searches with reduced affine gap extension parameters, which allowed large gaps up to 1000 bp to be traversed. Alignments between these 400 kb segments were generated using the parameters (−G 180 –E 1 –q 80 –r 30 –z 3 × 10-9 –Y 3 × 10-9 –e 1e-20 –F F). We discarded self-alignments, and wrote a list of perl scripts to reinsert the high-copy repeats back to these alignments. BLASTN results were parsed for alignments with length ≥1 kb and identity ≥88%. These initial seed alignments were subsequently reintroduced to create local alignments and then trimmed to define their end points. We then performed an optimal global alignment to generate accurate alignment statistics. Only alignments with length ≥ 1 kb and identity ≥ 90% were considered in our analysis.

### Whole-genome shotgun sequence detection of duplications (WSSD)

We used the WSSD strategy previously developed during the analysis of human genome [4] to assess duplication content in the silkworm. For a given genomic sequence, this method assesses depth-of-coverage and compares it with the average coverage depth. In regions of duplications, depth-of-coverage shows a statistically significant increase due to recruitment of paralogous reads. WSSD prefers to identify large SDs (≥10 kb in length, ≥ 94% sequence identity). We used two classes of sequences: (1) all finished BACs sequences deposited in GenBank; (2) whole silkworm genome sequence.

Firstly, short genome reads (<50 bp) and vector sequences were filtered out. After filtration, there were ~1.83 G clean reads left (size ranging from 52–964 bp long, ~4.5 converge of the genome) (Additional file 11). Each reference silkworm genome sequence masked for repeat sequences was compared by *Megablast* against the entire set of the silkworm whole-genome shotgun sequence reads (WGS, 3,810,411 reads). Our analysis was on the basis of a comparison of 3,810,411 WGS reads against the 432 Mb silkworm genome sequences. About 86.4% of (3,290,836) reads were remapped to the assembly. We used the following parameters (−D 3 –J F –P 93 –U T –F m –s 220), which allows for greed-algorithm extension into adjacent repetitive regions [6]. We wrote a perl script to detect every segment. Alignments were considered if they represented 90% of the reads with a rescored similarity of > 94%.

We used sliding window method in WSSD pipeline to calculate the reads depth (RD) value. Reads were firstly counted in overlapping (1 kb), sliding 5 kb windows. Initial calls were selected if six of seven or more sequential 5 kb overlapping windows had RD values that differ significantly from the average. Since the reads length varied significantly, the STDEV (~ 380) of the reads length was high in the silkworm. Furthermore, no segmental reference was previously reported in the silkworm, and it is impossible to identify the accurate RD value in SDs in the silkworm. And there is no information about a set of unique regions validated by FISH or other experiments. Thus, we removed the SDs regions identified by WGAC and 10 kb flanking regions of SDs. We defined significant alignment depth that greater than 3 standard deviations from the mean (Additional file 11). Only SD calls greater than 10 kb in length were kept in the final dataset. Because the silkworm strain *Dazao* (the sequenced strain of silkworm) is an experimental line and highly inbred, there is a reduced allelic variation in *Dazao*. We used a more sensitive metric for the detection of SDs [6]. This method increased sensitivity for detecting large single-duplications events (including recent, but low-frequency tandem duplications). In this way, we kept candidate segmental regions in which the divergent read (defined as those with identity higher than 99.8% aligned to the reference sequence with ratio higher than 0.5.

### Gene content

Gene content of the silkworm segmental duplications was assessed using the glean consensus gene set (http://silkworm.genomics.org.cn/) [17]. We obtained a total of 14,623 silkworm peptides from SilkDB. In addition, using Gene Ontology (GO) [50], we tested the whether the molecular function, biological process, and pathway terms were under- or overrepresented in SDs [24]. Pfam was also used to annotate the function of the genes in the SDs [51].

We also investigated the distribution of genes and segmental duplications on genomic sequences. It should be noted that a portion of genes in the silkworm have been not well-annotated or have been annotated with the designation “Unknown function”, which may result in the underestimation of the influence of genes in SDs.

### Quantitative real-time PCR (qRT-PCR) validation

Primer Premier 5.0 was used to design primers for qRT-PCR experiments (Additional file 12). Each PCR reaction was prepared as follows: 10 μl of SYBR-Green PCR master mix, 1 μl of each primer (10 μM), 7 μl of water, and 1 μl of genome template (whole genome DNA). Quantitative real-time PCR was carried out using the ABI Stepone plus system. The thermocycler program had an initial 95 denaturation step followed by 40 cycles consisting of a 10-s denaturation at 95, a 40-s annealing at 60, and a 30-s extension step at 72. At the end of each reaction, a disassociation curve was created, which was used to help to detect the presence of primer dimers of other unwanted amplification products that may produce a detectable cycle threshold (Ct) value.

We chose three regions (control_1, control_2, control_3) as controls for all qRT-PCR experiments, which represented single copy, 4 copies and TEs. Copy number was analyzed according to comparative Ct method. The Δ CT and ΔΔ CT were calculated by the formulas Δ CT = CT target – CT control (single copy) and ΔΔ CT = Δ CT SD samples - Δ CT single copy sample, respectively. To detect the accuracy of this method, we used the pipeline [52] to calculate the copy number of control_2, which was identified to be 4 copies *in silico*. The result showed that this gene was ~3.95 copies based on our method. Thus, it is reasonable to apply this pipeline to assess the segmental duplications.

## Competing interests

The authors declare that they have no competing interests.

## Authors’ contributions

ZZ designed the study. QZ performed the analyses and drafted the manuscript. MK provided the advice for the data analysis. SM provided the shotgun reads and BACs data, and read the manuscript. ZLZ provided help in the data analysis and revised the manuscript. ZZ supervised the study and revised the manuscript. All authors read and approved the final manuscript.

## Supplementary Material

Additional file 1: Table S1 The position information of the SDs in the silkworm genome. Click here for file

Additional file 2: Figure S1 (A) The silkworm SDs are enriched in the unassigned genome sequence (ChrUn). (B) Whole-genome interchromosomal alignments in the silkworm. Click here for file

Additional file 3: Figure S2 Examples of chromosomal distribution of detected SDs. SD distribution corresponded to blue bars. The red line showed the trend of the distribution of SDs. Click here for file

Additional file 4: Table S2 The genes in the SDs and their duplicated copies in the genome. Click here for file

Additional file 5: Table S3 The potential functions of the genes in the SDs identified by BLAST against *nr* database. Click here for file

Additional file 6: Table S4 The potential functions of the genes in the SDs predicting using Pfam. Click here for file

Additional file 7: Figure S3 Examples of whole-genome shotgun sequence detection (WSSD). The examples of reads mapped against a single region, a segmental duplication region, and a transposable element. The snapshot gave the number of reads mapping to the reference (single region, SDs and transposable elements). Blue lines indicate the reference region of single copy, SD and transposable element while the mapping reads were listed below. Click here for file

Additional file 8: Figure S4 SD content of genomes of different species in the phylogenetic tree. Click here for file

Additional file 9: Table S5 The comparison of the poor-annotated SD-content genes with other two Lepidoptera insects. Click here for file

Additional file 10: Figure S5 An example of the unannotated SD-content genes comparing to related species. Click here for file

Additional file 11: Figure S6 The short read distribution. (A) The number of reads with particular length. (B) The total size (Mb) of reads with particular length. (C) WSSD methods we used to identify the SDs in silkworm. Click here for file

Additional file 12: Table S6 Primer lists used in qPCR and BLAST validation of the control’s copy number and the BLAST validation of Con_1 and Con_2. Click here for file

## References

[B1] Eichler EE Sankoff D Structural dynamics of eukaryotic chromosome evolution Science 2003 301 5634 793 797 10.1126/science.1086132 12907789

[B2] Muller HJ Bar duplication Science 1936 83 2161 528 530 17806465 10.1126/science.83.2161.528-a

[B3] Ohno S Evolution by gene duplication 1970 New York: Springer-Verlag

[B4] Bailey JA Gu Z Clark RA Reinert K Samonte RV Schwartz S Adams MD Myers EW Li PW Eichler EE Recent segmental duplications in the human genome Science 2002 297 5583 1003 1007 10.1126/science.1072047 12169732

[B5] Cheng Z Ventura M She X Khaitovich P Graves T Osoegawa K Church D DeJong P Wilson RK Paabo S Rocchi M Eichler EE A genome-wide comparison of recent chimpanzee and human segmental duplications Nature 2005 437 7055 88 93 10.1038/nature04000 16136132

[B6] Bailey JA Church DM Ventura M Rocchi M Eichler EE Analysis of segmental duplications and genome assembly in the mouse Genome Res 2004 14 5 789 801 10.1101/gr.2238404 15123579 PMC479105

[B7] She X Cheng Z Zollner S Church DM Eichler EE Mouse segmental duplication and copy number variation Nat Genet 2008 40 7 909 914 10.1038/ng.172 18500340 PMC2574762

[B8] Nicholas TJ Cheng Z Ventura M Mealey K Eichler EE Akey JM The genomic architecture of segmental duplications and associated copy number variants in dogs Genome Res 2009 19 3 491 499 19129542 10.1101/gr.084715.108 PMC2661811

[B9] Sharp AJ Locke DP McGrath SD Cheng Z Bailey JA Vallente RU Pertz LM Clark RA Schwartz S Segraves R Oseroff VV Albertson DG Pinkel D Eichler EE Segmental duplications and copy-number variation in the human genome Am J Hum Genet 2005 77 1 78 88 10.1086/431652 15918152 PMC1226196

[B10] Graubert TA Cahan P Edwin D Selzer RR Richmond TA Eis PS Shannon WD Li X McLeod HL Cheverud JM Ley TJ A high-resolution map of segmental DNA copy number variation in the mouse genome PLoS Genet 2007 3 1 e3 10.1371/journal.pgen.0030003 17206864 PMC1761046

[B11] Redon R Ishikawa S Fitch KR Feuk L Perry GH Andrews TD Fiegler H Shapero MH Carson AR Chen W Cho EK Dallaire S Freeman JL Gonzalez JR Gratacos M Huang J Kalaitzopoulos D Komura D MacDonald JR Marsha CR Mei R Montgomery L Nishimura K Okamura K Shen F Somerville MJ Tchinda J Valsesia A Woodwark C Yang F Global variation in copy number in the human genome Nature 2006 444 7118 444 454 10.1038/nature05329 17122850 PMC2669898

[B12] Bailey JA Liu G Eichler EE An Alu transposition model for the origin and expansion of human segmental duplications Am J Hum Genet 2003 73 4 823 834 10.1086/378594 14505274 PMC1180605

[B13] Lupski JR Genomic disorders: structural features of the genome can lead to DNA rearrangements and human disease traits Trends Genet 1998 14 10 417 422 10.1016/S0168-9525(98)01555-8 9820031

[B14] Ji Y Eichler EE Schwartz S Nicholls RD Structure of chromosomal duplications and their role in mediating human genomic disorders Genome Res 2000 10 5 597 610 10.1101/gr.10.5.597 10810082

[B15] Samonte RV Eichler EE Segmental duplications and the evolution of the primate genome Nat Rev Genet 2002 3 1 65 72 11823792 10.1038/nrg705

[B16] Mita K Kasahara M Sasaki S Nagayasu Y Yamada T Kanamori H Namiki N Kitagawa M Yamashita H Yasukochi Y Kadono-Okuda K Yamamoto K Ajimura M Ravikumar G Shimomura M Nagamura Y Shin-I T Abe H Shimada T Morishita S Sasaki T The genome sequence of silkworm, *Bombyx mori* DNA Res 2004 11 1 27 35 10.1093/dnares/11.1.27 15141943

[B17] Xia Q Zhou Z Lu C Cheng D Dai F Li B Zhao P Zha X Cheng T Chai C Pan G Xu J Liu C Lin Y Qian J Hou Y Wu Z Li G Pan M Li C Shen Y Lan X Yuan L Li T Xu H Yang G Wan Y Zhu Y Yu M Shen W A draft sequence for the genome of the domesticated silkworm (*Bombyx mori*) Science 2004 306 5703 1937 1940.22 15591204 10.1126/science.1102210

[B18] Bailey JA Yavor AM Massa HF Trask BJ Eichler EE Segmental duplications: organization and impact within the current human genome project assembly Genome Res 2001 11 6 1005 1017 10.1101/gr.GR-1871R 11381028 PMC311093

[B19] Bickhart DM Hou Y Schroeder SG Alkan C Cardone MF Matukumalli LK Song J Schnabel RD Ventura M Taylor JF Garcia JF Tassell CP Sonstegard TS Eichler EE Liu GE Copy number variation of individual cattle genomes using next-generation sequencing Genome Res 2012 22 4 778 790 10.1101/gr.133967.111 22300768 PMC3317159

[B20] Sakudoh T Nakashima T Kuroki Y Fujiyama A Kohara Y Honda N Fujimoto H Shimada T Nakagaki M Banno Y Tsuchida K Diversity in copy number and structure of a silkworm morphogenetic gene as a result of domestication Genetics 2011 187 3 965 976 10.1534/genetics.110.124982 21242537 PMC3063685

[B21] D’Haene B Vandesompele J Hellemans J Accurate and objective copy number profiling using real-time quantitative PCR Methods 2010 50 4 262 270 10.1016/j.ymeth.2009.12.007 20060046

[B22] Fiston-Lavier AS Anxolabehere D Quesneville H A model of segmental duplication formation in *Drosophila melanogaster* Genome Res 2007 17 10 1458 1470 10.1101/gr.6208307 17726166 PMC1987339

[B23] She X Jiang Z Clark RA Liu G Cheng Z Tuzun E Church DM Sutton G Halpern AL Eichler EE Shotgun sequence assembly and recent segmental duplications within the human genome Nature 2004 431 7011 927 930 10.1038/nature03062 15496912

[B24] Liu GE Ventura M Cellamare A Chen L Cheng Z Zhu B Li C Song J Eichler EE Analysis of recent segmental duplications in the bovine genome BMC Genomics 2009 10 571 10.1186/1471-2164-10-571 19951423 PMC2796684

[B25] Tuzun E Bailey JA Eichler EE Recent segmental duplications in the working draft assembly of the brown Norway rat Genome Res 2004 14 4 493 506 10.1101/gr.1907504 15059990 PMC383293

[B26] Ai J Zhu Y Duan J Yu Q Zhang G Wan F Xiang ZH Genome-wide analysis of cytochrome P450 monooxygenase genes in the silkworm, *Bombyx mori* Gene 2011 480 1–2 42 50 21440608 10.1016/j.gene.2011.03.002

[B27] Sun W Shen YH Yang WJ Cao YF Xiang ZH Zhang Z Expansion of the silkworm GMC oxidoreductase genes is associated with immunity Insect Biochem Mol Biol 2012 42 12 935 945 10.1016/j.ibmb.2012.09.006 23022604

[B28] Zhang Y Dong Z Liu S Yang Q Zhao P Xia Q Identification of novel members reveals the structural and functional divergence of lepidopteran-specific Lipoprotein_11 family Funct Integr Genomics 2012 12 4 705 715 10.1007/s10142-012-0281-4 22534768

[B29] Vergara IA Mah AK Huang JC Tarailo-Graovac M Johnsen RC Baillie DL Chen N Polymorphic segmental duplication in the nematode *Caenorhabditis elegans* BMC Genomics 2009 10 329 10.1186/1471-2164-10-329 19622155 PMC2728738

[B30] Yu QY Lu C Li WL Xiang ZH Zhang Z Annotation and expression of carboxylesterases in the silkworm, *Bombyx mori* BMC Genomics 2009 10 533 10.1186/1471-2164-10-533 19930670 PMC2784812

[B31] Eichler EE Segmental duplications: what’s missing, misassigned, and misassembled–and should we care? Genome Res 2001 11 5 653 656 10.1101/gr.188901 11337463

[B32] Consortium TISG The genome of a lepidopteran model insect, the silkworm *Bombyx mori* Insect Biochem Mol Biol 2008 38 12 1036 1045 10.1016/j.ibmb.2008.11.004 19121390

[B33] Green P Against a whole-genome shotgun Genome Res 1997 7 5 410 417 9149937 10.1101/gr.7.5.410

[B34] Eichler EE Masquerading repeats: paralogous pitfalls of the human genome Genome Res 1998 8 8 758 762 9724321 10.1101/gr.8.8.758

[B35] Eichler EE Repetitive conundrums of centromere structure and function Hum Mol Genet 1999 8 2 151 155 10.1093/hmg/8.2.151 9931322

[B36] The BAC Resource Consortium Integration of cytogenetic landmarks into the draft sequence of the human genome Nature 2001 409 6822 953 958 10.1038/35057192 11237021 PMC7845515

[B37] Batzoglou S Jaffe DB Stanley K Butler J Gnerre S Mauceli E Berger B Mesirov JP Lander ES ARACHNE: a whole-genome shotgun assembler Genome Res 2002 12 1 177 189 10.1101/gr.208902 11779843 PMC155255

[B38] Bork P Copley R The draft sequences, Filling in the gaps Nature 2001 409 6822 818 820 10.1038/35057274 11236994

[B39] Osanai-Futahashi M Suetsugu Y Mita K Fujiwara H Genome-wide screening and characterization of transposable elements and their distribution analysis in the silkworm, *Bombyx mori* Insect Biochem Mol Biol 2008 38 12 1046 1057 10.1016/j.ibmb.2008.05.012 19280695

[B40] Gregory TR Synergy between sequence and size in large scale genomics Nature reviews 2005 6 699 708 10.1038/nrg1674 16151375

[B41] Bulet P Hetru C Dimarcq JL Hoffmann D Antimicrobial peptides in insects; structure and function Dev Comp Immunol 1999 23 4–5 329 344 10426426 10.1016/s0145-305x(99)00015-4

[B42] Zhao P Wang GH Dong ZM Duan J Xu PZ Cheng TC Xiang ZH Xia QY Genome-wide identification and expression analysis of serine proteases and homologs in the silkworm *Bombyx mori* BMC Genomics 2010 11 405 10.1186/1471-2164-11-405 20576138 PMC2996933

[B43] Asano N Yamashita T Yasuda K Ikeda K Kizu H Kameda Y Kato A Nash RJ Lee HS Ryu KS Polyhydroxylated alkaloids isolated from mulberry trees (*Morusalba L*.) and silkworms (*Bombyx mori L.*) J Agric Food Chem 2001 49 9 4208 4213 10.1021/jf010567e 11559112

[B44] Johnson ME Viggiano L Bailey JA Abdul-Rauf M Goodwin G Rocchi M Eichler EE Positive selection of a gene family during the emergence of humans and African apes Nature 2001 413 6855 514 519 10.1038/35097067 11586358

[B45] Paulding CA Ruvolo M Haber DA The Tre2 (USP6) oncogene is a hominoid-specific gene Proc Natl Acad Sci U S A 2003 100 5 2507 2511 10.1073/pnas.0437015100 12604796 PMC151371

[B46] Nei M Rooney AP Concerted and birth-and-death evolution of multigene families Annu Rev Genet 2005 39 121 152 10.1146/annurev.genet.39.073003.112240 16285855 PMC1464479

[B47] Thomas JH Rapid birth-death evolution specific to xenobiotic cytochrome P450 genes in vertebrates PLoS Genet 2007 3 5 e67 10.1371/journal.pgen.0030067 17500592 PMC1866355

[B48] Komoto N Quan GX Sezutsu H Tamura T A single-base deletion in an ABC transporter gene causes white eyes, white eggs, and translucent larval skin in the silkworm w-3(oe) mutant Insect Biochem Mol Biol 2009 39 2 152 156 10.1016/j.ibmb.2008.10.003 18996197

[B49] The International Silkworm Genome Consortium The genome of a lepidopteran model insect, the silkworm *Bombyx mori* Insect Biochem Mol Biol 2008 38 12 1036 1045 10.1016/j.ibmb.2008.11.004 19121390

[B50] Ye J Fang L Zheng H Zhang Y Chen J Zhang Z Wang J Li S Li R Bolund L WEGO: a web tool for plotting GO annotations Nucleic Acids Res 2006 34 Web Server issue W293 W297 16845012 10.1093/nar/gkl031 PMC1538768

[B51] Finn RD Mistry J Tate J Coggill P Heger A Pollington JE Gavin OL Gunasekaran P Ceric G Forslund K Holm L Sonnhammer ELL Eddy SR Bateman A The Pfam protein families database Nucleic Acids Res 2010 38 Database issue D211 D222 19920124 10.1093/nar/gkp985 PMC2808889

[B52] Zhang X Cheng T Wang G Yan Y Xia Q Cloning and evolutionary analysis of tobacco MAPK gene family Mol Biol Rep 2012 40 2 1407 1415 23079708 10.1007/s11033-012-2184-9

