# Supplementary figures and images for: Segmental duplications in the silkworm genome
Source: BMC Genomics. 2013 Jul 31;14:521. doi: 10.1186/1471-2164-14-521 (PMC3735471; doi:10.1186/1471-2164-14-521)

(A)

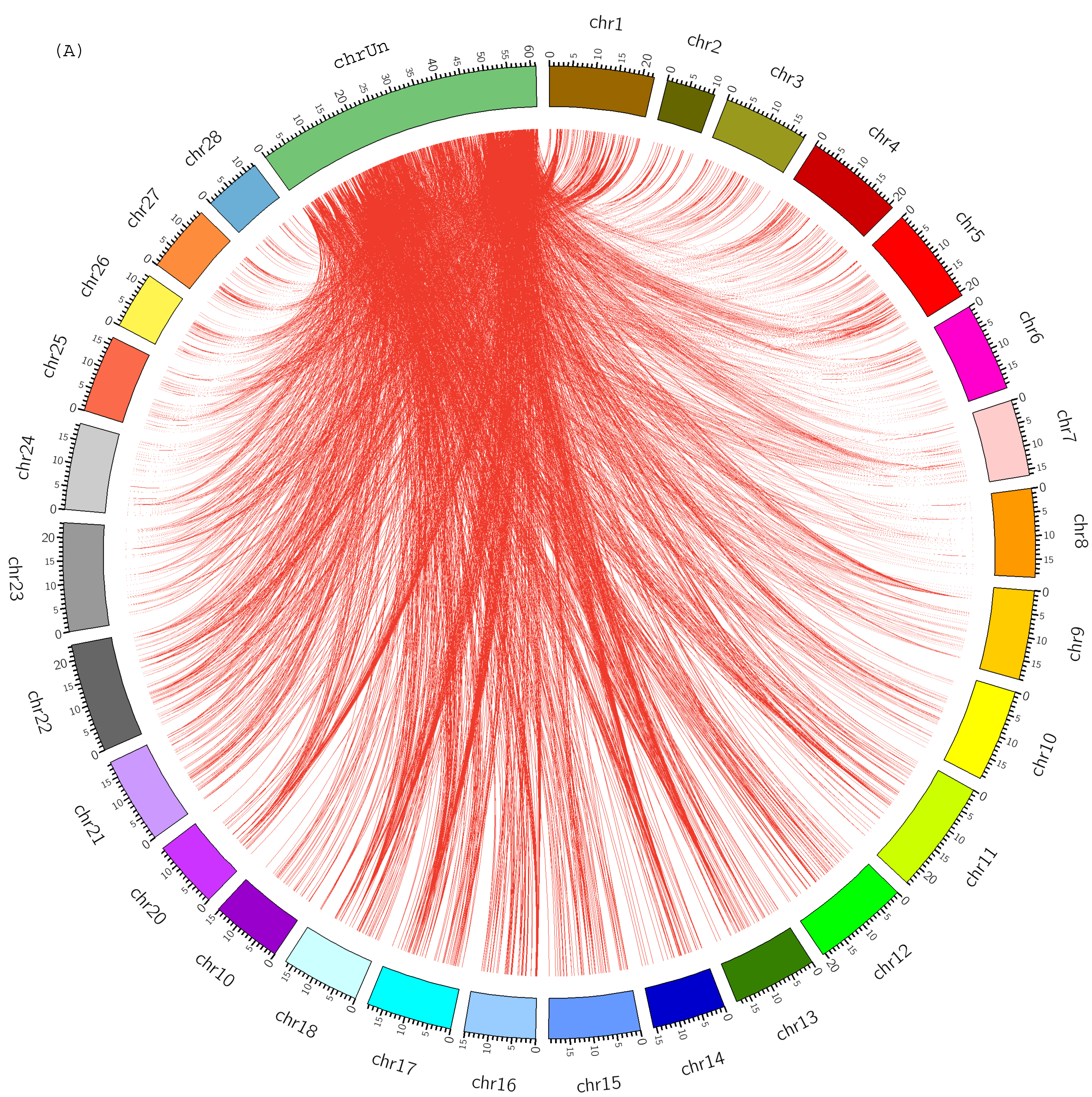

(B)

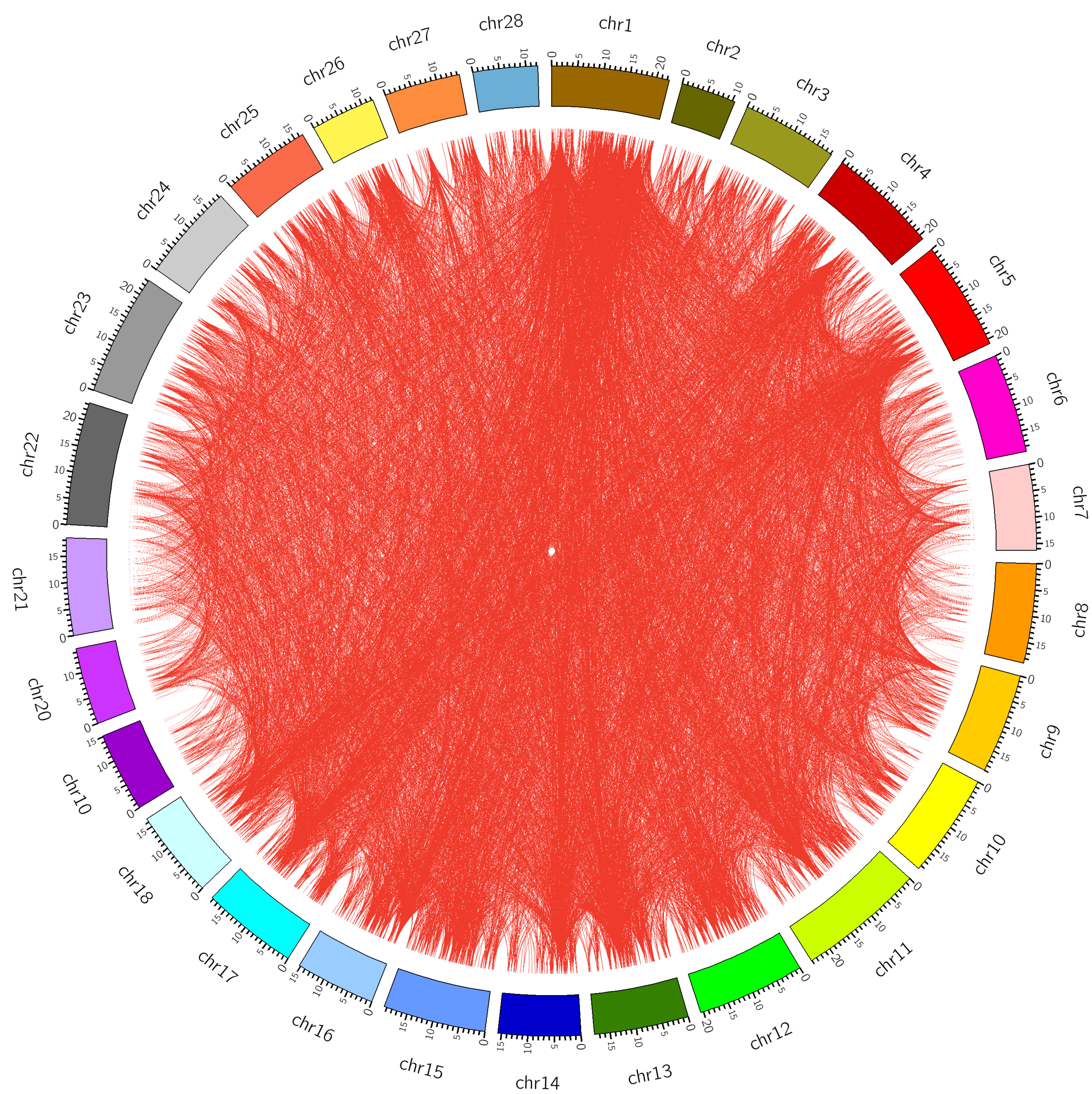

Supplement: Additional file 2: Figure S1 — (A) The silkworm SDs are enriched in the unassigned genome sequence (ChrUn). (B) Whole-genome interchromosomal alignments in the silkworm. [file 1471-2164-14-521-S2.pdf]

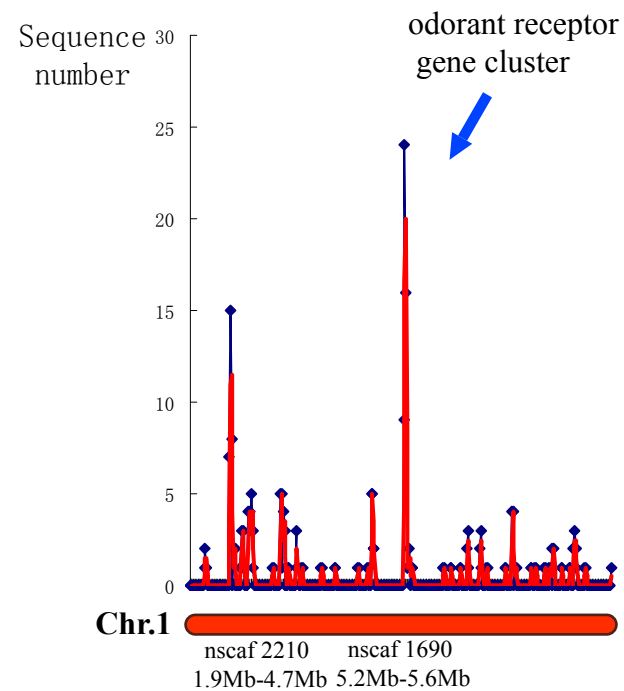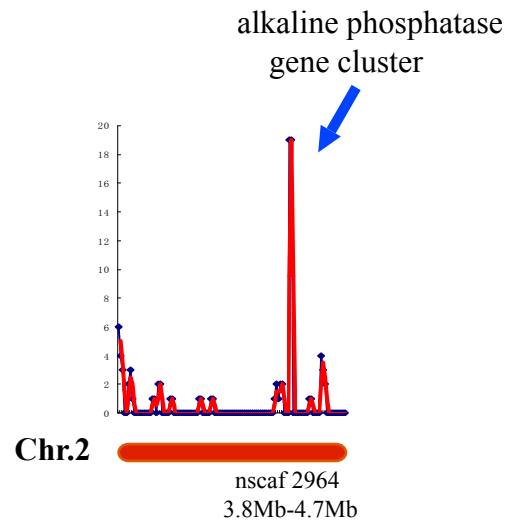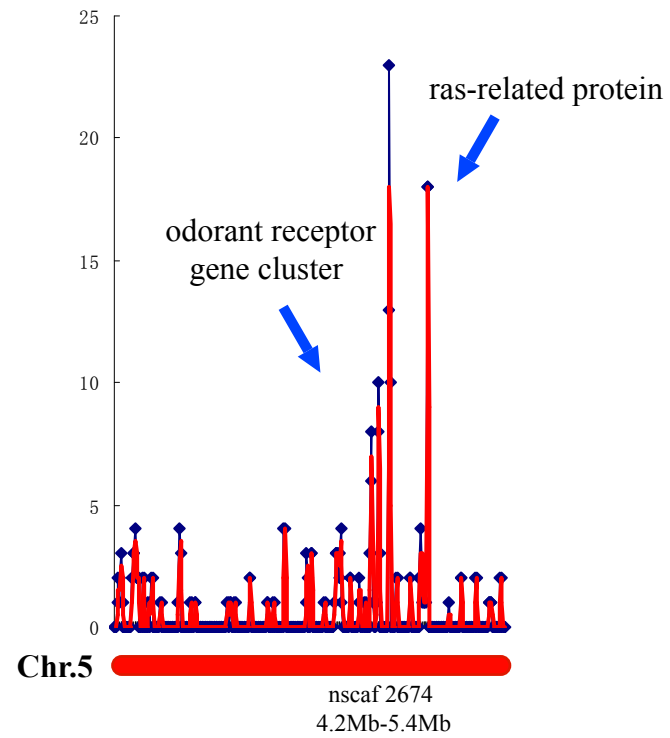

Supplement: Additional file 3: Figure S2 — Examples of chromosomal distribution of detected SDs. SD distribution corresponded to blue bars. The red line showed the trend of the distribution of SDs. [file 1471-2164-14-521-S3.pdf]

Reads depth

transposable elements

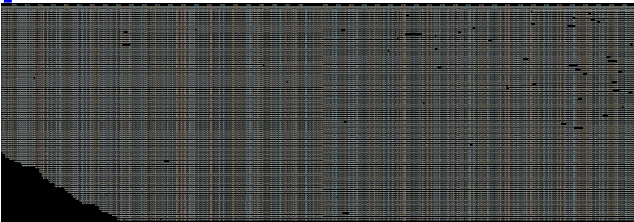

single copy in genome

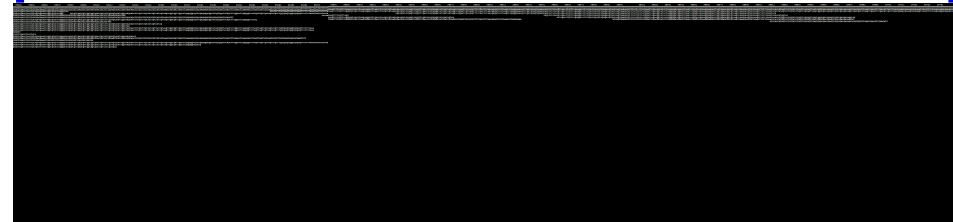

segmental duplications

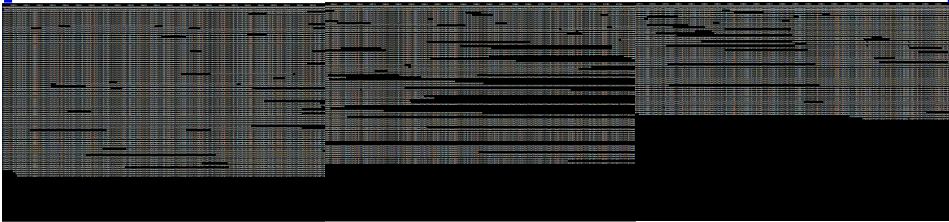

Supplement: Additional file 7: Figure S3 — Examples of whole-genome shotgun sequence detection (WSSD). The examples of reads mapped against a single region, a segmental duplication region, and a transposable element. The snapshot gave the number of reads mapping to the reference (single region, SDs and transposable elements). Blue lines indicate the reference region of single copy, SD and transposable element while the mapping reads were listed below. [file 1471-2164-14-521-S7.pdf]

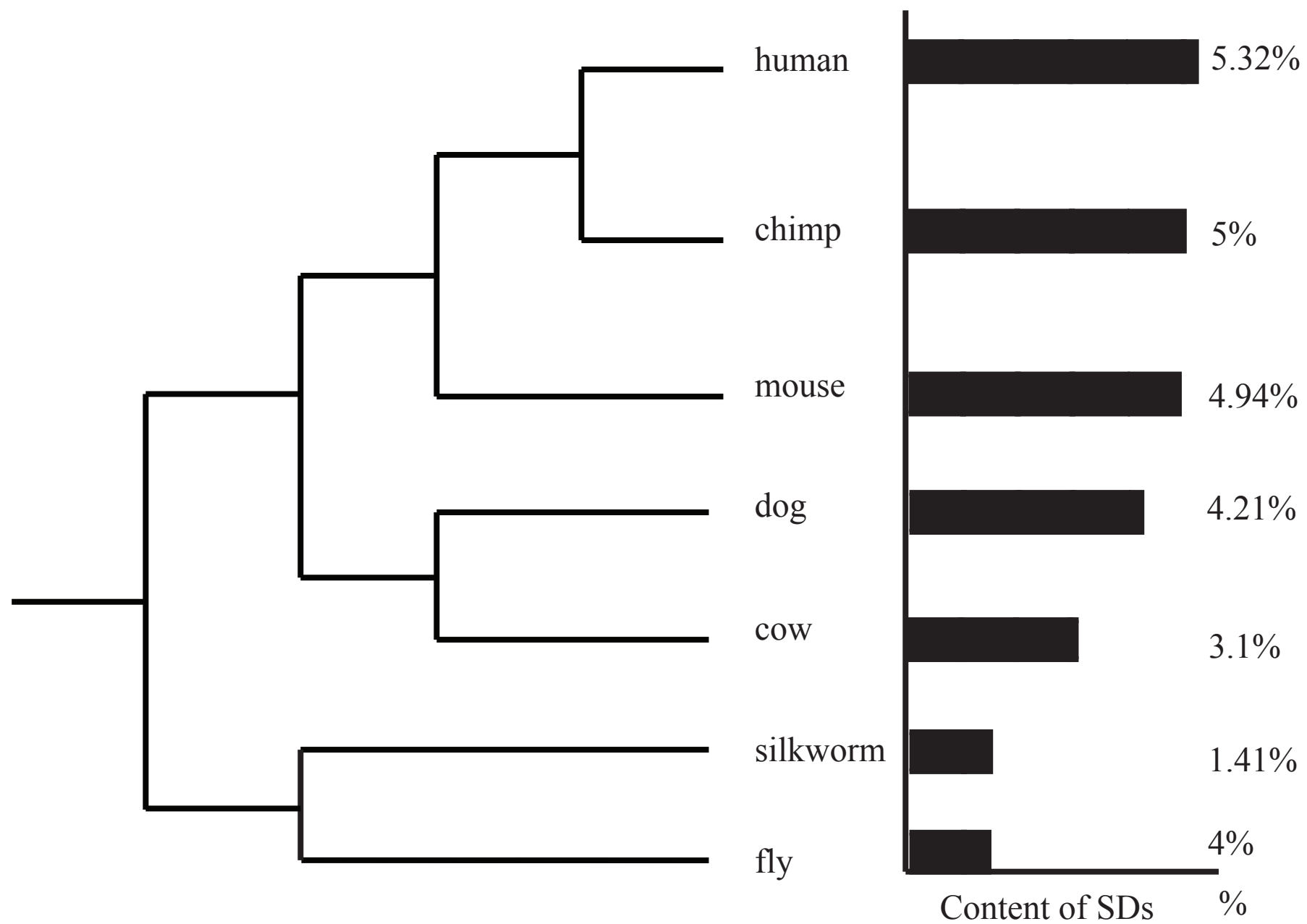

Supplement: Additional file 8: Figure S4 — SD content of genomes of different species in the phylogenetic tree. [file 1471-2164-14-521-S8.pdf]
