# Supplementary material for: Segmental duplications in the silkworm genome
Source: BMC Genomics. 2013 Jul 31;14:521. doi: 10.1186/1471-2164-14-521 (PMC3735471; doi:10.1186/1471-2164-14-521)

Table 1S Primer lists used in qPCR and BLAST validation of the control’ copy numbers.

| **GeneBank accession number** | **Length of products(bp)** | **Primer Sequence** | ***T*_M_(℃)** |
| --- | --- | --- | --- |
| AB159446.1 | 152 | GGCAGGGAACTATCACACCG | 60.1 |
|  |  | AGTCTCCCACCACTACGCAAG | 59.2 |
| AP009014.1 | 173 | GAACAATTTCCGCCAAGACAC | 57.8 |
|  |  | TAAGATGCTTTCACGCTGGAT | 59 |
| AP009015.1 | 228 | GCATACGTGGCATGGGATAAT | 58.9 |
|  |  | TGGGAGACTAGACCTGCAAAAG | 59.7 |
| AP009017.1 | 99 | ACGAGTAGTAGATGGCGGTGTC | 58.6 |
|  |  | CTCCCCGTTACTATTCAGTTTGT | 57.5 |
| AP009018.1 | 101 | GTGGGGTTGATGTCTATTCGC | 59.4 |
|  |  | TACTCGTGTAGGAAGATGTCGC | 57.3 |
| AP009021.1 | 197 | CACGCACACATTTTTTTGTATTC | 58.1 |
|  |  | CTTATTTATGGCCTTATTTTTCG | 56.2 |
| AP009022.1 | 174 | GTTTTCACGGCATAGGCATTC | 69.9 |
|  |  | TAGCACTTTCACTGACACAATCTTC | 58.5 |
| AP008992.1 | 106 | AACCACATGGCCTCGGATT | 69.8 |
|  |  | TTGCTGAGTCTGCTCGTCTTG | 60.1 |
| AP008996.1 | 107 | CTTTTCGTGGGGTTGATGTCT | 59.1 |
|  |  | TACTCGTGTAGGAAGATGTCGC | 57.3 |
| AP009006.1 | 152 | GCTTCTTCCATTTGACTCCATAC | 60.0 |
|  |  | ATTCCTCAGAGCACTCCCCAT | 69.8 |
| Con_1 | 157 | ATTTTACCACATCTGGCGACTT | 57.6 |
|  |  | GATTGCTACCATCACCTTTCTCAT | 57.7 |
| Con_2 | 188 | GACCTCTATCCCCACTCCATC | 57.3 |
|  |  | CTGTCTGCTGCCTTGCTTTCT | 59.9 |
| Con_3 | 100 | TAACATCCGCTTCTTTCCACA | 58.4 |
|  |  | AAACTTCTACCCGTCCAACTCA | 58.3 |


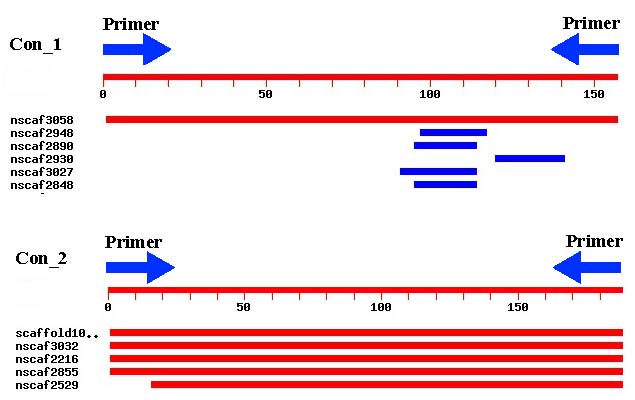

Supplement: Additional file 4: Table S2 — The genes in the SDs and their duplicated copies in the genome. [file 1471-2164-14-521-S4.doc]
