# Supplementary material for: Segmental duplications in the silkworm genome
Source: BMC Genomics. 2013 Jul 31;14:521. doi: 10.1186/1471-2164-14-521 (PMC3735471; doi:10.1186/1471-2164-14-521)
Supplement: Additional file 6: Table S4 — The potential functions of the genes in the SDs predicting using Pfam. [file 1471-2164-14-521-S6.doc]

Table 3S The potential functions of genes in SDs identified by BLAST against *nr* database.

| **GENE** | **Position** | **Exons** | **Best Hits** | **Identity** |
| --- | --- | --- | --- | --- |
| BGIBMGA007347-TA | [nscaf2883:1699129..1681128 (- strand)](http://silkworm.genomics.org.cn/cgi-bin/gbrowse/silkdb?name=nscaf2883:1699129..1681128) | 8 | olfactory receptor | 96 |
| BGIBMGA013241-TA | [nscaf3063:1664775..1666777 (+ strand)](http://www.silkdb.org/cgi-bin/gbrowse/silkdb?name=nscaf3063:1664775..1666777) | 2 | P450 | 99 |
| BGIBMGA008164-TA | [nscaf2891:7730..9278 (- strand)](http://www.silkdb.org/cgi-bin/gbrowse/silkdb?name=nscaf2891:7730..9278) | 2 | Lipoprotein_11 | 92 |
| BGIBMGA008824-TA | [nscaf2925:150717..153317 (+ strand)](http://www.silkdb.org/cgi-bin/gbrowse/silkdb?name=nscaf2925:150717..153317) | 5 | membrane-bound alkaline phosphatase | 84 |
| BGIBMGA013237-TA | [nscaf3063:1606752..1609201 (+ strand)](http://www.silkdb.org/cgi-bin/gbrowse/silkdb?name=nscaf3063:1606752..1609201) | 2 | cytochrome P450 | 96 |
| BGIBMGA009573-TA | [nscaf2962:998093..999370 (- strand)](http://www.silkdb.org/cgi-bin/gbrowse/silkdb?name=nscaf2962:998093..999370) | 1 | Lipoprotein_11 | 84 |
| BGIBMGA010168-TA | [nscaf2986:1478755..1480285 (- strand)](http://www.silkdb.org/cgi-bin/gbrowse/silkdb?name=nscaf2986:1478755..1480285) | 2 | Lipoprotein_11 | 94 |
| BGIBMGA004881-TA | [nscaf2818:2885484..2886744 (+ strand)](http://www.silkdb.org/cgi-bin/gbrowse/silkdb?name=nscaf2818:2885484..2886744) | 3 | bmp-2 | 84 |
| BGIBMGA007598-TA | [nscaf2888:9732609..9737765 (- strand)](http://www.silkdb.org/cgi-bin/gbrowse/silkdb?name=nscaf2888:9732609..9737765) | 7 | GABA-gated ion channel | 90 |
| BGIBMGA011770-TA | [nscaf3031:853850..856100 (+ strand)](http://www.silkdb.org/cgi-bin/gbrowse/silkdb?name=nscaf3031:853850..856100) | 3 | G protein pathway suppressor 1 | 100 |
| BGIBMGA014429-TA | [scaffold644:11381..13100 (+ strand)](http://www.silkdb.org/cgi-bin/gbrowse/silkdb?name=scaffold644:11381..13100) | 5 | serine protease | 80 |
| BGIBMGA012386-TA | [nscaf3041:569984..571630 (+ strand)](http://www.silkdb.org/cgi-bin/gbrowse/silkdb?name=nscaf3041:569984..571630) | 2 | P450 | 92 |
| BGIBMGA013298-TA | [nscaf3066:677503..724891 (- strand)](http://www.silkdb.org/cgi-bin/gbrowse/silkdb?name=nscaf3066:677503..724891) | 38 | Ryanodine receptor | 81 |
| BGIBMGA003566-TA | [nscaf2674:1720302..1723064 (- strand)](http://www.silkdb.org/cgi-bin/gbrowse/silkdb?name=nscaf2674:1720302..1723064) | 4 | serine protease | 96 |
| BGIBMGA007327-TA | [nscaf2882:233359..236693 (+ strand)](http://www.silkdb.org/cgi-bin/gbrowse/silkdb?name=nscaf2882:233359..236693) | 4 | antennal-enriched UDP-glycosyltransferase | 86 |
| BGIBMGA003461-TA | [nscaf2674:6836132..6850048 (- strand)](http://www.silkdb.org/cgi-bin/gbrowse/silkdb?name=nscaf2674:6836132..6850048) | 16 | hypothetical protein KGM_11046 | 81 |
| BGIBMGA005831-TA | [nscaf2838:2457249..2458118 (+ strand)](http://silkworm.swu.edu.cn/cgi-bin/gbrowse/silkdb?name=nscaf2838:2457249..2458118) | 1 | alcohol dehydrogenase | 93 |
| BGIBMGA014313-TA | [scaffold1329:3832..4977 (- strand)](http://silkworm.swu.edu.cn/cgi-bin/gbrowse/silkdb?name=scaffold1329:3832..4977) | 2 | protein KGM_14113 | 89 |
| BGIBMGA010406-TA | [nscaf2993:5249081..5261842 (- strand)](http://silkworm.swu.edu.cn/cgi-bin/gbrowse/silkdb?name=nscaf2993:5249081..5261842) | 20 | argonaute 2 | 93 |
| BGIBMGA013635-TA | [nscaf3079:918217..925844 (+ strand)](http://silkworm.swu.edu.cn/cgi-bin/gbrowse/silkdb?name=nscaf3079:918217..925844) | 6 | FK506-binding protein | 100 |
| BGIBMGA013270-TA | [nscaf3063:3057112..3057864 (+ strand)](http://silkworm.swu.edu.cn/cgi-bin/gbrowse/silkdb?name=nscaf3063:3057112..3057864) | 1 | 1 3-dehydroecdysone 3alpha-reductase | 86 |
| BGIBMGA008818-TA | [nscaf2925:44134..46448 (- strand)](http://silkworm.swu.edu.cn/cgi-bin/gbrowse/silkdb?name=nscaf2925:44134..46448) | 5 | membrane-bound alkaline phosphatase | 86 |
| BGIBMGA013236-TA | [nscaf3063:1596046..1599190 (+ strand)](http://silkworm.swu.edu.cn/cgi-bin/gbrowse/silkdb?name=nscaf3063:1596046..1599190) | 3 | P450 | 82 |
| BGIBMGA010877-TA | [nscaf3005:17431..18357 (+ strand)](http://silkworm.swu.edu.cn/cgi-bin/gbrowse/silkdb?name=nscaf3005:17431..18357) | 1 | Lipoprotein_11 | 92 |
| BGIBMGA011001-TA | [nscaf3013:1329154..1338945 (+ strand)](http://silkworm.swu.edu.cn/cgi-bin/gbrowse/silkdb?name=nscaf3013:1329154..1338945) | 8 | aminoacylase | 89 |
| BGIBMGA005593-TA | [nscaf2829:101779..106939 (- strand)](http://silkworm.swu.edu.cn/cgi-bin/gbrowse/silkdb?name=nscaf2829:101779..106939) | 4 | putative signal recognition particle 68 kDa protein | 94 |
| BGIBMGA014428-TA | [scaffold644:923..2605 (- strand)](http://silkworm.swu.edu.cn/cgi-bin/gbrowse/silkdb?name=scaffold644:923..2605) | 5 | serine protease precursor | 90 |
| BGIBMGA004376-TA | nscaf2795:2873837..2876518 (- strand) | 6 | Proteasome | 88 |
| BGIBMGA014115-TA | nscaf463:114103..122823 (- strand) | 8 | beta-N-acetylglucosaminidase isoform A | 99 |
| BGIBMGA009621-TA | nscaf2962:1000811..1002755 (+ strand) | 2 | putative paralytic peptide-binding protein | 97 |
| BGIBMGA014524-TA | scaffold769:10251..11882 (- strand) | 5 | serine protease precursor | 91 |
| BGIBMGA000837-TA | nscaf1898:16163449..16165672 (- strand) | 3 | antennal esterase | 97 |
| BGIBMGA002771-TA | nscaf2575:4412208..4421213 (- strand) | 10 | CUB | 81 |
| BGIBMGA004034-TA | nscaf2767:818655..839449 (- strand) | 4 | PREDICTED: similar to CG13229-PA | 83 |
| BGIBMGA013423-TA | nscaf3072:2525028..2563582 (+ strand) | 32 | RhoGAP | 84 |
| BGIBMGA003656-TA | nscaf2674:2968892..2977567 (+ strand) | 7 | cystathionine gamma-lyase | 83 |
| BGIBMGA011000-TA | nscaf3013:1319845..1324681 (+ strand) | 9 | aminoacylase | 97 |
| BGIBMGA000965-TA | nscaf1898:8084128..8093948 (- strand) | 8 | PREDICTED: similar to CG12218-PA | 81 |
| BGIBMGA013239-TA | nscaf3063:1630051..1631436 (+ strand) | 1 | cytochrome P450 | 85 |
| BGIBMGA011002-TA | nscaf3013:1341740..1347880 (+ strand) | 9 | aminoacylase | 95 |
| BGIBMGA002340-TA | nscaf2330:3959629..3965504 (+ strand) | 6 | regulatory subunit A of protein phosphatase 2, alpha isoform | 92 |
| BGIBMGA012555-TA | nscaf3048:204154..205695 (+ strand) | 1 | H+ transporting ATP synthase beta subunit isoform 2 | 93 |
| BGIBMGA014116-TA | nscaf463:79310..96865 (- strand) | 10 | beta-N-acetylglucosaminidase isoform A | 99 |
| BGIBMGA014550-TA | scaffold798:17961..22613 (+ strand) | 4 | AF482926_1 acyl-CoA desaturase PintIPAE | 99 |
| BGIBMGA002426-TA | nscaf2511:1997297..2012111 (- strand) | 10 | alpha 1,2-mannosidase | 82 |
| BGIBMGA010302-TA | nscaf2987:37990..41540 (- strand) | 2 | gustatory receptor 18 | 86 |
| BGIBMGA013861-TA | nscaf3098:985586..986422 (+ strand) | 1 | antennal-enriched UDP-glycosyltransferase | 100 |
| BGIBMGA013160-TA | nscaf3063:3048950..3049702 (- strand) | 1 | AF255341_1 3-dehydroecdysone 3alpha-reductase | 89 |
| BGIBMGA008165-TA | nscaf2891:11977..13242 (+ strand) | 1 | putative paralytic peptide-binding protein | 96 |
| BGIBMGA012385-TA | nscaf3041:544340..546381 (+ strand) | 2 | cytochrome P450 | 99 |
| BGIBMGA014619-TA | scaffold950:2562..9040 (+ strand) | 6 | estrogen sulfotransferase | 98 |
| BGIBMGA005832-TA | nscaf2838:2460302..2461171 (+ strand) | 1 | alcohol dehydrogenase | 89 |
| BGIBMGA012603-TA | nscaf3052:434945..436464 (+ strand) | 4 | CU36A_MANSE Pupal cuticle protein 36a precursor | 91 |
| BGIBMGA014546-TA | scaffold795:1233..2378 (+ strand) | 2 | hypothetical protein KGM_14113 | 89 |
| BGIBMGA003474-TA | nscaf2674:6337776..6341779 (- strand) | 7 | casein kinase 2 alpha subunit | 94 |
| BGIBMGA010876-TA | nscaf3005:14053..15607 (- strand) | 2 | putative paralytic peptide-binding protein | 93 |
| BGIBMGA009788-TA | nscaf2964:3814768..3820151 (+ strand) | 3 | antennal-enriched UDP-glycosyltransferase | 97 |
| BGIBMGA008793-TA | nscaf2916:637210..638436 (+ strand) | 2 | unnamed protein product | 97 |
| BGIBMGA003376-TA | nscaf2655:101277..112063 (- strand) | 10 | PREDICTED: similar to myotubularin related protein 9 | 80 |
| BGIBMGA002465-TA | nscaf2511:998508..1003088 (+ strand) | 5 | nuclear factor NF-kappa-B p110 subunit isoform 1 | 100 |
| BGIBMGA002486-TA | nscaf2511:1967012..1981521 (+ strand) | 10 | alpha 1,2-mannosidase | 83 |
| BGIBMGA010204-TA | nscaf2986:1481475..1482737 (+ strand) | 2 | putative paralytic peptide-binding protein | 92 |
| BGIBMGA006971-TA | nscaf2865:5459094..5465427 (- strand) | 7 | estrogen sulfotransferase | 84 |
| BGIBMGA014315-TA | scaffold1340:1623..4924 (- strand) | 3 | PREDICTED: similar to bagpipe homeobox homolog 1 | 94 |
