# Supplementary material for: Segmental duplications in the silkworm genome
Source: BMC Genomics. 2013 Jul 31;14:521. doi: 10.1186/1471-2164-14-521 (PMC3735471; doi:10.1186/1471-2164-14-521)
Supplement: Additional file 10: Figure S5 — An example of the unannotated SD-content genes comparing to related species. [file 1471-2164-14-521-S10.pdf]

(A)

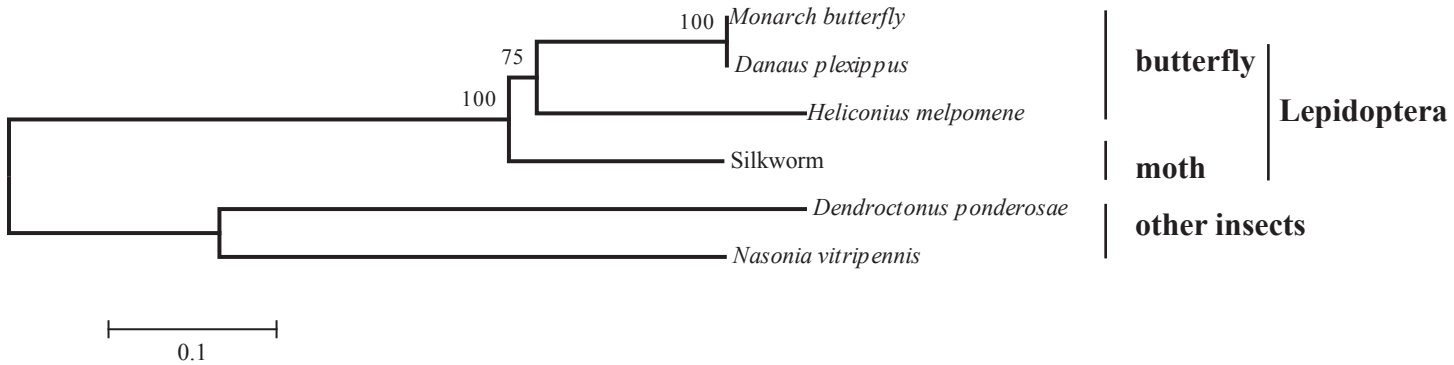
100
Monarch butterfly
75
Danaus plexippus
100
Heliconius melpomene
Silkworm
Dendroctonus ponderosae
Nasonia vitripennis
butterfly
moth
other insects
Lepidoptera
0.1

(B)

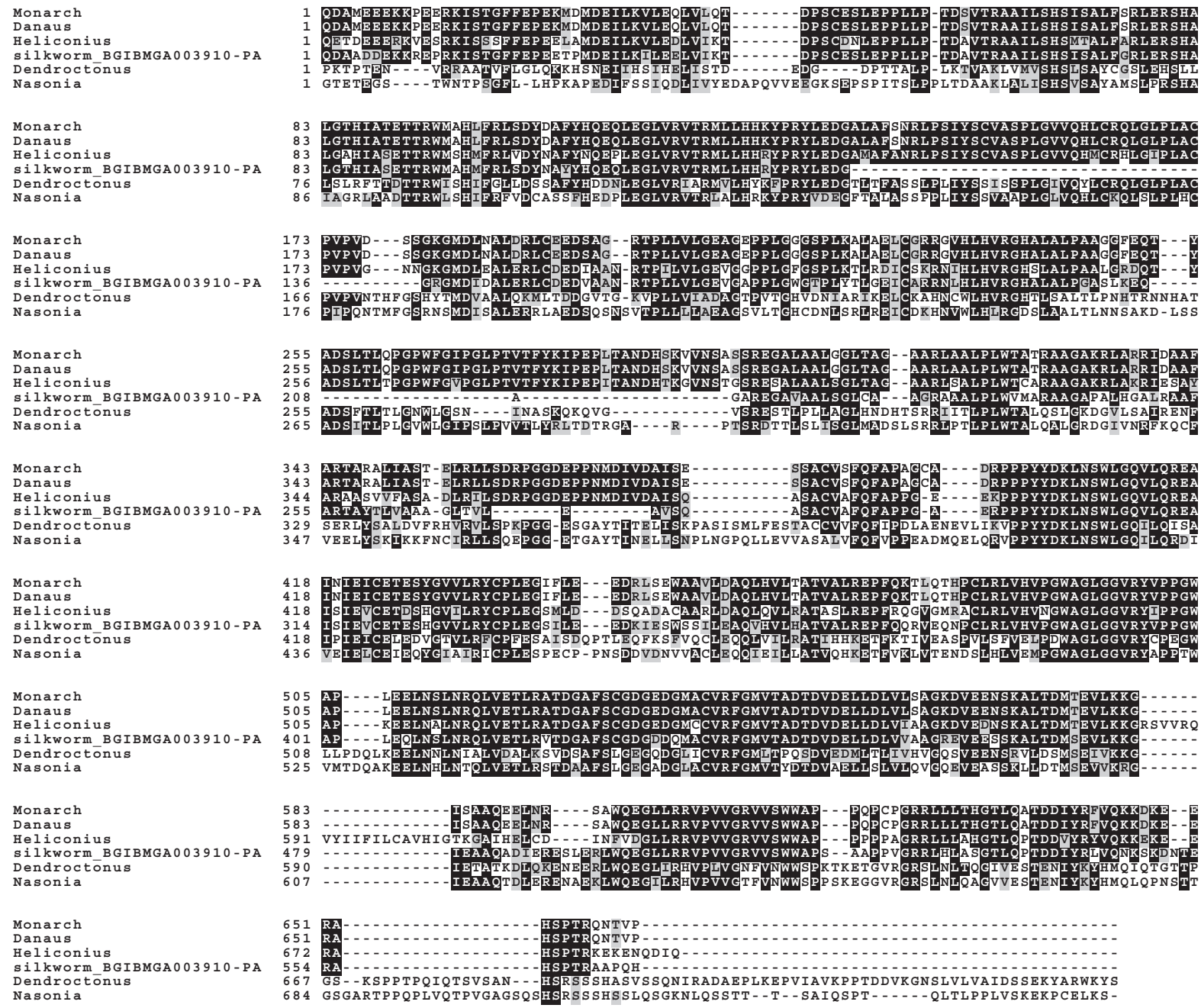
Monarch 1 QDAMEEEKKPEERKISTGFFEPEKMDMDEILKVLEQLVLQT-------DPSCESLEPPLLP-TDSVTRAAILSHSISALFSRLERSHA
Danaus 1 QDAMEEEKKPEERKISTGFFEPEKMDMDEILKVLEQLVLQT-------DPSCESLEPPLLP-TDSVTRAAILSHSISALFSRLERSHA
Heliconius 1 QETDEEERKVESRKISSSFFEPEELAMDEILKVLEDLVIKT-------DPSCDNLEPPLLP-TDAVTRAAILSHSMTALFARLERSHA
silkworm_BGIBMGA003910-PA 1 QDAADDEKKREPRKISTGFFEPEETPMDEILKILEELVIKT-------DPSCESLEPPLLP-TDAVTRAAILSHSISALFGRLERSHA
Dendroctonus 1 PKTPTEN----VRRAATVFLGLQKKHSNEITHSIHELISTD------EDG----DPTTALP-LKTVAKLVMVSHSLSAYCGSLEHSLL
Nasonia 1 GTETEGS----TWNTPSGFL-LHPKAPEDIFSSIQDLIVYEDAPQVVEEGKSEPSPITSLPPLTDAAKLALISHSVSAYAMSLPRSHA
Monarch 83 LGTHIATETTRWMAHLFRLSDYDAFYHQEQLEGLVRVTRMLLHHKYPRYLEDGALAFSNRLPSIYSCVASPLGVVQHLCRQLGLPLAC
Danaus 83 LGTHIATETTRWMAHLFRLSDYDAFYHQEQLEGLVRVTRMLLHHKYPRYLEDGALAFSNRLPSIYSCVASPLGVVQHLCRQLGLPLAC
Heliconius 83 LGAHIASETTRWMSHMFRLVDYNAFYNQEPLEGLVRVTRMLLHHRYPRYLEDGAMAFANRLPSIYSCVASPLGVVQHMCRHLGIPLAC
silkworm_BGIBMGA003910-PA 83 LGTHIASETTRWMAHMFRLSDYNAYYHQEQLEGLVRVTRMLLHHRYPRYLEDG----------
Dendroctonus 76 LSLRFTTDTTRWISHIFGLLDSSAFYHDDNLEGLVRIARMVLHYKFPRYLEDGTLTFASSLPLIYSSISSPLGIVQYLCRQLGLPLAC
Nasonia 86 IAGRLAADTTRWLSHIFRFVDCASSFHEDPLEGLVRVTRLALHRKYPRYVDEGFTALASSPPLIYSSVAAPLGLVQHLCKQLSLPLHC
Monarch 173 PVPVD---SSGKGMDLNALDRLCEEDSAG--RTPLLVLGEACEPPLGGGSPLKALAELCGRRGVHLHVRGHALALPAAGGFEQT---Y
Danaus 173 PVPVD---SSGKGMDLNALDRLCEEDSAG--RTPLLVLGEACEPPLGGGSPLKALAELCGRRGVHLHVRGHALALPAAGGFEQT---Y
Heliconius 173 PVPVG---NNGKGMDLEALERLCDEDIAAN-RTPILVLGEVCGPPLGFGSPLKTLRDICSKRNIHLHVRGHSLALPAALGRDQT---Y
silkworm_BGIBMGA003910-PA 136 ----------CRGMDIDALERLCDEDVAAN-RTPLLVLGEVCAPPLGWGTPLYTLGEICARRNLHLHVRGHALALPGASLKEQ-----
Dendroctonus 166 PVPVNTHFGSHYTMDVAALQKMLTDDGVTG-KVPLLVIADAGTPVTGHVDNIARIKELCKAHNCWLHVRGHTLSALTLPNHTRNNHAT
Nasonia 176 PIPQNTMFGSRNSMDISALERRLAEDSQSNSVTPLLLLAEAGSVLTGHCDNLSRLREICDKHNVWLHLRGDSLAALTLNNSAKD-LSS
Monarch 255 ADSLTLQPGPWFGIPGLPTVTFYKIPEPLTANDHSKVVNSASSREGALAALGGLTAG--AARLAALPLWTATRAAGAKRLARRIDAAF
Danaus 255 ADSLTLQPGPWFGIPGLPTVTFYKIPEPLTANDHSKVVNSASSREGALAALGGLTAG--AARLAALPLWTATRAAGAKRLARRIDAAF
Heliconius 256 ADSLTLTPGPWFGVPGLPTVTFYKIPEPITANDHTKGVNSTGSRESALAALSGLTAG--AARLSALPLWTCARAAGAKRLAKRIESAY
silkworm_BGIBMGA003910-PA 208 ----------A----------GAREGAVAALSGLCA---AGRAAALPLWVMARAAGAPALHGALRAAF
Dendroctonus 255 ADSFTLTLGNWLGSN----INASKQKQVG----------VSRESTLPLLAGLHNDHTSRRIITLPLWTALQSLGKDGVLSATRENF
Nasonia 265 ADSITLPLGVWLGIPSLPVVTLYRLTDTRGA----R----PTSRDTTLSLISGLMADSLSRRLPTLPLWTALQALGRDGIVNRFKQCF
Monarch 343 ARTARALIAST-ELRLLSDRPGGDEPPNMDIVDAISE----------SSACVSFQFAPAGCA----DRPPPYYDKLNSWLGQVLQREA
Danaus 343 ARTARALIAST-ELRLLSDRPGGDEPPNMDIVDAISE----------SSACVSFQFAPAGCA----DRPPPYYDKLNSWLGQVLQREA
Heliconius 344 ARAASVVFASA-DLRILSDRPGGDEPPNMDIVDAISQ----------ASACVAFQFAPPG-E----EKPPPYYDKLNSWLGQVLQREA
silkworm_BGIBMGA003910-PA 255 ARTAYTLVAAA-GLTVL-------E--------AVSQ----------ASACVAFQFAPPG-A----ERPPPYYDKLNSWLGQVLQREA
Dendroctonus 329 SERLYSALDVFRHVRVLSPKPGG-ESGAYTITELISKPASISMLFESTACCVVFQFTPDLAENEVLIKVPPYYDKLNSWLGQILQISA
Nasonia 347 VEELYSKIKKFNCIRLLSQEPGG-ETGAYTINELLSNPLNGPQLLEVVASALVFQFVPPEADMQELQRVPPYYDKLNSWLGQILQRDI
Monarch 418 INIEICETESYGVVLRYCPLEGIFLE---EDRLSEWAAVLDAQLHVLTATVALREPFQKTLQTHPCLRLVHVPGWAGLGGVRYVPPGW
Danaus 418 INIEICETESYGVVLRYCPLEGIFLE---EDRLSEWAAVLDAQLHVLTATVALREPFQKTLQTHPCLRLVHVPGWAGLGGVRYVPPGW
Heliconius 418 ISIEVCETDSHGVILRYCPLEGSMLD---DSQADACAARLDAQLQVLRATASLREPFRQGVGMRACLRLVHVNGWAGLGGVRYIPPGW
silkworm_BGIBMGA003910-PA 314 ISIEVCETESHGVVLRYCPLEGSILE---EDKIESWSSILEAQVHVLHATVALREPFQQRVEQNPCLRLVHVPGWAGLGGVRYVPPGW
Dendroctonus 418 IPIEICELEDVGTVLRFCPFESAISDQPTLEQFKSFVQCLEQQLVILRATIHHKETFKTIVEASPVLSFVEIPDWAGLGGVRYCPEGW
Nasonia 436 VEIELCEIEQYGIAIRICPLESPECP-PNSDDVDNVVACLEQQIEILLATVQHKETFVKLVTENDSLHLVEMPGWAGLGGVRYAPPTW
Monarch 505 AP----LEELNSLNRQLVETLRATDGAFSCGDGEDGMACVRFGMVTADTDVDELLDLVLSAGKDVEENSKALTDMTEVLKKG------
Danaus 505 AP----LEELNSLNRQLVETLRATDGAFSCGDGEDGMACVRFGMVTADTDVDELLDLVLSAGKDVEENSKALTDMTEVLKKG------
Heliconius 505 AP----KEELNALNRQLVETLRATDGAFSCGDGEDGMCCVRFGMVTADTDVDELLDLVIAAGKDVEDNSKALTDMTEVLKKGRSVVRQ
silkworm_BGIBMGA003910-PA 401 AP----LEQLNSLNRQLVETLRVTDGAFSCGDGDDQMACVRFGMVTADTDVDELLDLVVAAGREVEESSKALTDMSEVLKKG------
Dendroctonus 508 LLPDQLKEELNNLNIALVDALKSVDSAFSLGEGQDGLICVRFGMLTPQSDVEDMLTLIVHVGQSVEENSRVLDSMSEIVKKG------
Nasonia 525 VMTDQAKEELNHLNTQLVETLRSTDAAFSLGEGADGLACVRFGMVTYDTDVAELLSLVLQVGQEVEASSKLLDTMSEVVKRG------
Monarch 583 -------------ISAAQEELNR----SAWQEGLLRRVPVVGRVVSWWAP---PQPCPGRRLLLTHGTLQATDDIYRFVQKKDKE--E
Danaus 583 -------------ISAAQEELNR----SAWQEGLLRRVPVVGRVVSWWAP---PQPCPGRRLLLTHGTLQATDDIYRFVQKKDKE--E
Heliconius 591 VYIIFILCAVHIGTKGAIHELCD----INFVDGLLRRVPVVGRVVSWWAP---PPPPAGRRLLLAHGTLQPTDDVYRYVQKKEKE--E
silkworm_BGIBMGA003910-PA 479 -------------IEAAQADIERESLERLWQEGLLRRVPVVGRVVSWWAPS--AAPPVGRRLHLASGTLQPTDDIYRLVQNKSKDNTE
Dendroctonus 590 -------------IETATKDLQKENEERLWQEGLIRHVPLVGNFVNWWSPKTKETGVRGRSLNLTQGIVESTENIYKYHMQIQTGTTP
Nasonia 607 -------------IEAAQTDLERENAEKLWQEGLIRHVPVVGTFVNWWSPPSKEGGVRGRSLNLQAGVVESTENIYKYHMQLQPNSTT
Monarch 651 RA----------HSPTRQNTVP
Danaus 651 RA----------HSPTRQNTVP
Heliconius 672 RA----------HSPTRKEKENQDIQ
silkworm_BGIBMGA003910-PA 554 RA----------HSPTRAAPQH
Dendroctonus 667 GS--KSPPTPQIQTSVSAN---HSRSSSHASVSSQNIRADAEPLKEPVIAVKPPTDDVKGNSLVLVAIDSSEKYARWKYS
Nasonia 684 GSGARTPPQPLVQTPVGAGSQSHSRSSSHSSLQSGKNLQSSTT--T--SAIQSPT------QLTLPPLVSKEKPCELKS-
