# Supplementary material for: Segmental duplications in the silkworm genome
Source: BMC Genomics. 2013 Jul 31;14:521. doi: 10.1186/1471-2164-14-521 (PMC3735471; doi:10.1186/1471-2164-14-521)
Supplement: Additional file 11: Figure S6 — The short read distribution. (A) The number of reads with particular length. (B) The total size (Mb) of reads with particular length. (C) WSSD methods we used to identify the SDs in silkworm. [file 1471-2164-14-521-S11.pdf]

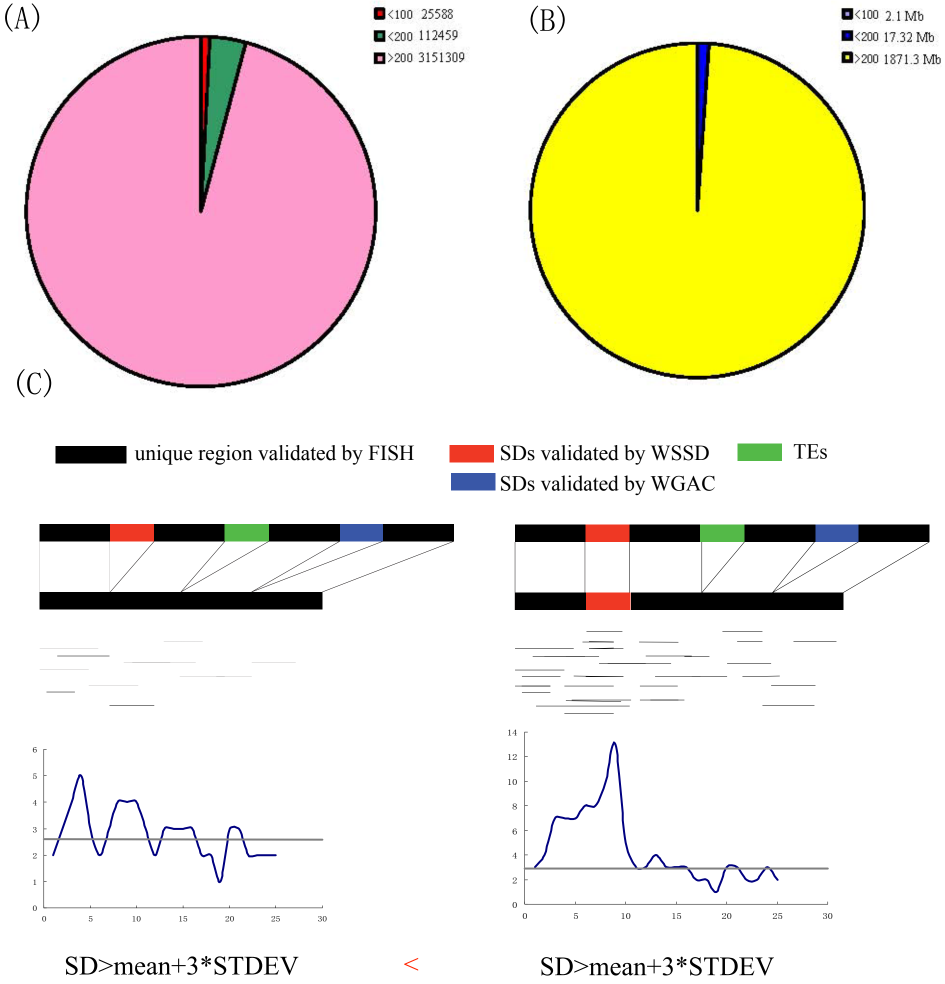

(A)
<100 25588
<200 112459
>200 3151309
(B)
<100 2.1 Mb
<200 17.32 Mb
>200 1871.3 Mb
(C)
unique region validated by FISH
SDs validated by WSSD
TEs
SDs validated by WGAC
SD>mean+3*STDEV
<
SD>mean+3*STDEV
